# Extracellular vesicles from human adipose-derived stem cell spheroids: Characterization and therapeutic implications in diabetic wound healing

**DOI:** 10.1016/j.mtbio.2024.101333

**Published:** 2024-11-08

**Authors:** Edgar Daniel Quiñones, Mu-Hui Wang, Kuan-Ting Liu, Ting-Yu Lu, Guan-Yu Lan, Yu-Ting Lin, Yu-Liang Chen, Tang-Long Shen, Pei-Hsun Wu, Yu-Sheng Hsiao, Er-Yuan Chuang, Jiashing Yu, Nai-Chen Cheng

**Affiliations:** a Taiwan International Graduate Program, Sustainable Chemical Science & Technology, Academia Sinica, Institute of Chemistry, Taipei 115, Taiwan; b Department of Surgery, National Taiwan University Hospital and College of Medicine, Taipei 100, Taiwan; c Department of Chemical Engineering, National Taiwan University, Taipei 10617, Taiwan; d Program in Materials Science and Engineering, University of California San Diego, CA, 92093, USA; e Department of Plant Pathology & Microbiology, National Taiwan University, Taiwan; f Johns Hopkins Institute for NanoBioTechnology, John Hopkins University Baltimore, MD 21218, USA; g Department of Materials Science and Engineering, National Taiwan University of Science and Technology, Taipei 106, Taiwan; h School of Biomedical Engineering, Taipei Medical University, Taipei 11031, Taiwan; i Research Center for Developmental Biology and Regenerative Medicine, National Taiwan University, Taipei, Taiwan

**Keywords:** Adipose-derived stem cell (ASC), Cell spheroids, Cell sheet, Extracellular vesicles, Diabetes wound healing

## Abstract

The management of diabetic wounds presents a considerable challenge within the realm of clinical practice. Cellular-derived nanoparticles, or extracellular vesicles (EV), generated by human adipose-derived stem cells (hASCs) have been investigated as promising candidates for the treatment of diabetic wounds. Nevertheless, limitations on the yield, as well as the qualitative angiogenic properties of the EV produced, have been a persistent issue. In this study, a novel approach involving the use of various cell culture morphologies, such as cell spheroids, on hASC was used to promote both EV yield and qualitative angiogenic properties for clinical use, with an emphasis on the in vivo angiogenic properties exhibited by the EV. Moreover, an increase in the secretion of the EV was confirmed after cell spheroid culture. Furthermore, microRNA(miRNA) analysis of the produced EVs indicated an increase in the presence of wound healing-associated miRNAs on the cell spheroid EV. Analysis of the effectiveness of the treated EVs in vitro indicated a significant promotion of the biological function of fibroblast and endothelial cells, cell migration, and cell proliferation post-cell spheroid EV application. Meanwhile, in vivo experiments on diabetic rats indicated a significant increase in collagen production, re-epithelization, and angiogenesis of the diabetic wound after EV administration. In this investigation, we posit that the use of cell spheroids for the culture of hASC represents a novel approach to enhance the substantial secretion of extracellular vesicles while increasing the angiogenic wound healing properties. This innovation holds promise for augmenting the therapeutic potential of EVs in diabetic wound healing, aligning with the exigencies of clinical applications for these nanoparticles.

## Graphical abstract

**Figure:**
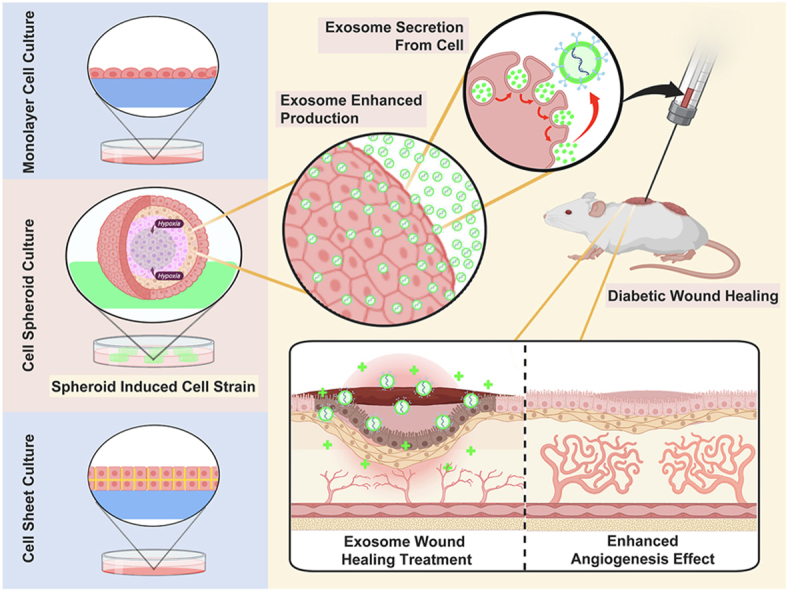

## 1 Introduction

Wound healing is a highly complex and crucial process subdivided into a variety of steps and involving countless mediators, reactions, and growth factors, which can be broadly grouped into four phases: hemostasis, inflammation, proliferation, and tissue remodeling or resolution [1,2]. Maintaining and balancing such a multitude of reactions is astonishingly difficult, especially in situations in which the patient suffers from chronic, long-term diseases. One such disease is diabetes mellitus, a chronic health condition affecting millions of people all over the world, in which the production and/or effectiveness of insulin are compromised. Such a sharp decline in the effective production of insulin directly affects the self-repairing capacity of diabetes patients [3,4]. These so-called diabetic wounds are characterized as being highly morbid, presenting a challenge for their treatment contributing to the high percentage of diabetes patients that present a variety of long-term wounds. This percentage has been estimated to be as high as 34 % in the case of lower extremities. Due to the various underlying biochemical disorders that a diabetes patient presents, treatment of these wounds poses a yet-to-be-overcome challenge. These disorders can range from hallmarks such as lack of sustained growth for productive granulated tissue, reduced tissue oxygenation, numbness in the area, and alterations of sympathetic function, among others. The combined effect of these disorders leads to a significant number of diabetes patients, as much as 39.4 % by some estimates, who are ultimately required to undergo lower extremity amputations. Even after the amputation, diabetes patients present a 55 % greater risk of death compared to non-diabetic patients. As an indication of this phenomenon, amputations secondary to a diabetic foot ulcer are associated with a five-year survival rate as low as 31 % for significant limb amputees [5,6]. These diabetes-associated wound complications have emerged as important clinical problems, but current treatments have been proven limited in their effectiveness.

The continuation of these attenuating circumstances has led to the development of a variety of strategies aimed at promoting diabetic wound healing, ranging from the use of growth factors and skin substitutes to the application of acellular dermal matrices. However, the application of these strategies has been held back due to inadequate outcomes, instances of rejection, and elevated costs [7]. Consequently, significant resources have been dedicated into developing alternative strategies such as stem cell manipulation, which has seen a multitude of advances, in particular their impact on the enhancement of wound healing and other angiogenesis related mechanisms, as a unique approach to this issue.

Mesenchymal stem cells (MSCs), which typically possess the ability to divide into more stem cells while maintaining their innate multi-lineage differentiation potential, have demonstrated their effectiveness in treating diabetic wounds. In particular, adipose-derived stem cells have shown to be an advantageous option from a clinical perspective, as these can be harvested with relative ease and safety in abundant numbers through liposuction. Human adipose-derived stem cells (hASC) contained in the isolate whole fat are obtained by a series of filtrations, collagenase digestion, and centrifugation of the adipose tissue to obtain the cellular fraction used in regenerative medicine [8,9]. Furthermore, it has been found that the concentration of stem cells yielded from adipose tissue is over 500 times larger than those found in other stem cell sources, such as bone marrow [[10], [11], [12]]. hASCs have been found capable of promoting human proliferation by both directly contacting cells and paracrine activation in wound healing. However, recent studies have indicated that the intrinsic mechanism responsible for the effects of MSCs is more strongly linked to their paracrine effects instead of their ability to straightforwardly differentiate into the injured cell [[13], [14], [15]]. These paracrine effects are strongly related to the release of extracellular vesicles from hASCs while at the same time minimizing the risk of rejection taken when hASCs are directly implanted into the wound area.

Extracellular vesicles (EVs) are membrane-derived vesicles released by cells under physiological and pathological conditions. EVs can be subdivided into microvesicles, exosomes, and apoptotic bodies. Each of these subgroups can be differentiated based on origin or physiological characteristics such as biogenesis, release pathways, size, content, and function. The capability of EVs to carry and deliver such sensitive biomolecules is one of the most interesting characteristics of these cellular structures. These can serve as vehicles to transfer a variety of bioactive cargoes such as protein, messenger RNA (mRNA), and micro-RNA (miRNA) into recipient cells, invoking biological changes of gene expression, proliferation, and differentiation of the recipient cells [[16], [17], [18], [19]].

The content, or cargo, of EVs not only mimics the composition of the donor cell type of origin, but most notably, the cargos of EVs are highly subjected to changes during the culturing/growth phase of the cells and can be influenced by the local microenvironment, pH, physical or mechanical stress, and a multiplicity of other external stimuli [[20], [21], [22]]. Currently, a diverse number of research projects have indicated that the efficiency of EV secretion, both qualitative and quantitative, can be enhanced by preconditioning the cells. This has been thus far achieved through genetic manipulation, hypoxia conditioning, intracellular calcium addition, and treatment with bioactive molecules [[22], [23], [24], [25], [26]]. One of the strategies that has garnered a great amount of attention due to its enhancement of both the quantity and quality of EVs produced from a cell culture is the use of three-dimensional cell culturing methods (cell spheroids, cell sheets, etc.). A number of studies have demonstrated that MSCs cultured in 3D spheroids, exhibit an increase in trophic factor secretion and higher therapeutic potential when compared to MSCs in a monolayer culture (such as interleukin-11, VEGF, bFGF, and angiogenin). Furthermore, various studies have suggested that MSCs in 3D spheroids are self-activated increasing prostaglandin E2 (PGE2) secretion thus enhancing the anti-inflammatory characteristics of the cells [[27], [28], [29], [30], [31]]. Although numerous explanations have been extended to account for these significant enhancements, the presence of a hypoxic microenvironment in the central core of the MSCs spheroids coupled with the increase of intracellular stress response has been the driving force for the increased secretion. Therefore, the 3D spheroid culture of MSCs may be a valid alternative to increase the production of therapeutically enhanced active MSCs [28].

In the present study, the effects of various cell culture techniques (monolayer, cell sheet, and cell spheroid) on the qualitative and quantitative characteristics of EVs derived from human adipose-derived stem cells (hASC) were analyzed, with a special focus on the enhancements of EV production and angiogenic potential. Critical parameters of EVs, such as size, yield, and genetic content, were tested, after which in-vitro experiments were carried out to measure their uptake capacity and angiogenic potential. Finally, in-vivo experiments using diabetic rat models corroborated the angiogenic capabilities of the EVs produced from the different cell culturing techniques.

## 2 Materials and methods

### 2.1 Cell culture

The hASCs were obtained from a combination of several nondiabetic donors as described previously [32,33]. Briefly, subcutaneous adipose tissue was finely minced and placed in a digestion solution containing 1 mg/ml collagenase type I (Gibco). Following digestion, the cell suspension was filtered and cultured in an expansion medium comprising Dulbecco's modified Eagle's medium (DMEM)/F-12 (Hyclone), 10 % fetal bovine serum (FBS; Biological Industries), 1 % penicillin-streptomycin (Biological Industries). The cells were cultured at 37 °C in a 5 % CO_2_ incubator, and the medium was changed every two to three days. On reaching 90 % confluence, the cells are detached with 0.05 % trypsin-EDTA (Biological Industries) and replated, and the third- and fourth-passage hASCs were used for different experiments. The various hASC cell cultures used henceforth were expanded until the cell confluency in the plate, or flask reached values approximating 90 % before they were used in the assorted cell morphology culture techniques.

### 2.2 Sheet formation of hASCs

To fabricate the desired cell sheets, fourth-passage hASCs cells previously mentioned were seeded with a density of approximately 2.5x10^4^ cells/cm^2^ as previously described [32,34,35]. The culture medium consisted of DMEM, high glucose (DMEM-HG; Gibco), 10 % FBS, 1 % penicillin-streptomycin, and 250 μM ascorbate 2-phosphate (A2-P, Sigma) cultured for seven days. The culture medium was refreshed every two to three days. Then cells were washed with phosphate-buffered saline (PBS; Corning) twice and changed medium to DMEM-HG with 10 % EV-depleted FBS (Gibco) for three days, after which the follow-up experiments and EV purification experiments were carried out.

### 2.3 Spheroid formation of hASCs

Our previous report showed that hASC spheroids of similar size could be fabricated in a high-throughput fashion by adjusting the cell seeding density and the surface topography of the micropatterned agarose structures designated as microwells [32], after which the agarose microwells containing the cells are centrifuged to facilitate and assure the formation of tight and uniformly formed cell spheroids. From the applied external force, hASCs in each concave pit will aggregate to form spheroids with identical morphology and diameters, guaranteeing homogeneity in the subsequent test. In more detail, monolayer hASCs were cultured in a T75 cell culture flask until 90 % cell confluency was reached. Following this, hASC cells were removed from the flask and added at a concentration of 2000, 4000, or 8000 cells per spheroid microwell, depending on the desired experimental conditions. Each of the three-dimensional petri-dish agarose molds contained a total of 256 microwells for the formation of spheroids. After hASC cells were added, the mold was centrifuged at 900 rpm for 5 min to accelerate the formation of the spheroids. After spheroid formation, these were cultured in the mold for one day and then dispersed by a solution of 1:1 Trypsin and accutase, and further cultured on a 10 cm dish with the density of 4 x 10^5^/dish for three days (culture medium for monolayer and spheroids were DMEM-HG (Gibco), 10 % FBS, 1 % penicillin-streptomycin). After three days of cell culture, these cells were washed by PBS (Corning) twice, and their medium changed to DMEM-HG with 10 % EV-depleted FBS (Gibco) for three days, after which the follow-up experiments and EV purification experiments were carried out.

### 2.4 hASC characterization

hASC were characterized after growth under two different cell culture morphologies (cell sheet and cell spheroid) via cell surface antigen expression and differentiation assays. Previous to the flow cytometry of the hASC, samples were incubated with the following antibodies: human monoclonal antibodies against CD31 (BD pharmingen), CD34, CD44, CD73, CD90 (all from BioLegend) and CD166 (BioLegend). The samples were analyzed using a flow cytometer (FACScan; Becton Dickinson). Positive cells were determined as the proportion of the population with higher fluorescence than 95 % of the isotype control. hASCs were then cultured in DMEM-HG supplemented with 10 % FBS. When the cells reached 80 % confluence, the medium was changed to a respective induction medium. Adipogenic differentiation was induced by DMEM-HG supplemented with 10 % FBS, 1 % penicillin/streptomycin, 500 mM 3-isobutyl-1-methylxanthine, 1 mM dexamethasone, 10 mM insulin, and 400 mM indomethacin (all from Sigma). Osteogenic differentiation was induced by DMEM-HG supplemented with 10 % FBS, 1 % penicillin/streptomycin, 10 nM dexamethasone, 50 mM A2-P, 10 nM 1a,25-dihydroxyvitamin D3, and 10 mM b-glycerophosphate (all from Sigma). hASCs were fixed in 4 % paraformaldehyde and stained with Oil Red O (Sigma) for the adipogenesis assay or Alizarin Red S (Sigma) for the osteogenesis assay on day 14. Moreover, adipogenic and osteogenic differentiation was induced and examined by histology as previously described [33].

### 2.5 Isolation of EVs

Following the collection of medium from the hASC cells cultured, for a total of 1x10^6^ cells for each cell experiment and isolation, under different cell culture morphologies, the medium was initially centrifuged at 500×*g* for 10 min to remove large cell debris, after which the supernatant was collected and centrifuged once again at 1000×*g* for 15 min to remove the largest cellular apoptotic vesicles. Following this, the supernatant was transferred into a Macrosep centrifugal Filter Unit (MWCO = 100 kDa; Pall life sciences) and centrifuged at 5000×*g* for 30 min. Then the concentrated medium was transferred into a Microsep centrifugal Filter Units (MWCO = 100 kDa; Pall life sciences) and centrifuged at 7500×*g* for 10 min to obtain the final concentrated EV-rich medium. Following this, the concentrated EV-rich medium was run through a size exclusion chromatography column (qEV original; Izon) with a setup size ranging from 50 nm to 200 nm, in which the fractions to be recovered were set to have a volume of 0.4 ml for a total fraction number of seven. The desired fractions are then recovered and stored for future use. The elution buffer used in the size exclusion chromatography column was salt deficient PBS.

### 2.6 Physical characterization of EVs

The characterization of EVs was carried out via NanoSight LM10 (Malvern Instruments; Malvern, UK) with Nanoparticle Tracking Analysis (NTA) software. Both size distribution and concentration were evaluated, with each group sample measured three times with a camera level set at fourteen and acquisition time of 30 s. An estimated range of 20–100 objects per frame with more than 200 completed tracks were analyzed for each video. The detection threshold was set at the beginning of each sample and kept constant for each repeat. The total surface protein concentration of the EVs was measured by BCA, using a BCA Protein Assay Kit.

### 2.7 Protein and uptake characterization of EVs

EVs were resuspended in PBS and spotted onto Formvar-coated grids for 1 min. Adsorbed EVs were fixed in 2 % paraformaldehyde for 5 min at room temperature and washed with distilled water. Then, EVs were either negatively stained using 2 % uranyl acetate or stained with a specific antibody for 1 min. The prepared EV slides were examined with a Hitachi H-7650 transmission electron microscope (Hitachi). In addition, the size, concentration, and particle size distribution of EVs were identified by NanoSight NS300 and Nanoparticle Tracking Analysis software (NanoSight).

Purified microvesicles were lyzed in cell lysis buffer (RayBiotech). The selected proteins were further analyzed by Western blot. The primary antibody used for immunoblotting included anti-CD9 (cell signaling), anti-CD63 (Abcam) and anti-ALIX. After extensive washing, the membranes were further incubated with horseradish peroxidase-conjugated secondary antibodies for 1 h. The blots were then developed using an enhanced chemiluminescence detection system (Millipore).

PKH26 dye (Sigma) was diluted in diluent C to a final concentration of 8 μM (dye solution). Then 10 μg of EVs from the monolayer or spheroid in 20 μL PBS were diluted with 80 μL diluent C, added to the dye solution, and incubated for 5 min while mixed with gentle pipetting. Excess dye was bound with 100 μL 10 % EV-depleted fetal bovine serum (Hyclone) in Dulbecco's modified Eagle's medium (Hyclone). Then the EVs were diluted to 1 mL with PBS and pelleted by ultracentrifugation at 100,000×*g* for 70 min at 4 °C (Type 90 Ti; Beckman Coulter). The pellet was gently resuspended in 50 μL EGM2 with a supplement (PromoCell). The PKH26-labeled EVs were then cultured on human umbilical vein endothelial cells (HUVECs) (Lonza) for 24 h. After fixation, the EVs were washed three times with PBS prior to immunostaining with 4 % formaldehyde. Then, the cytoskeleton was stained with phalloidin (BIOlegend) for 45 min at room temperature prior to three washes with PBS for 5 min each. After the final wash, 4′,6-diamidino-2-phenylindole (DAPI; 1:10000) (BIOlegend) in PBS was added, and cells were stained for 10 min before imaging via microscopy.

### 2.8 RNA and miRNA data analysis

Exosomal RNA was isolated by the RNeasy MinElute Cleanup Kit. (Qiagen, Germany). After isopropanol precipitation and ethanol wash, RNA quantity and purity were assessed at 260 nm and 280 nm using a BioDrop μLITE (BioDrop). Steps involving library preparation and sequencing were carried out according to the manufacturer's protocol from Illumina. Library construction of all samples was used by QIAseq miRNA Library Kit (Qiaqen). Raw sequences were obtained from the Illumina NovaSeq 6000 and expected to generate 3M (million reads or Gb) per sample. RNA-seq analysis went through a filtering process to obtain qualified reads. Trimmomatics was implemented to trim or remove the reads according to the quality score. The micro-RNA(miRNA) expression level was calculated as RPM (Reads Per Million). The reference miRNA annotations were retrieved from miRBase 22.1. miRNA regulated was determined with fold change ≧ 2.0 or ≦ −2.0.

To discern the differentially expressed miRNAs between the monolayer and sheet or monolayer and spheroid, we applied a criterion of more than a two-fold change (i.e., log(FC) > 1). Moreover, within the miRNAs displaying a two-fold difference, those with counts lower than twenty in both comparing conditions were excluded. This step aimed to alleviate the potential overestimation of high fold changes in miRNA genes with low expression levels. The TAM 2.0 (PMID: 29878154) was used for gene enrichment analysis to identify the associated functions from the identified miRNA gene list.

### 2.9 Scratch assay

HS68 fibroblast cells were plated onto 12-well plates (1 × 10^5^ cells/well) in DMEM-HG supplemented with 10 % FBS and 1 % penicillin-streptomycin. When the cells reached 90 % cell confluence, the cells were removed from the plate using a 1-mL pipette tip following a straight line and washed thrice with PBS. Thereafter, the cells were exposed to DMEM-HG supplemented with 10 % EV-free FBS and 1 % penicillin-streptomycin with the various hASC EV groups (monolayers, cell sheet, and cell spheroids), all with a concentration of 50 μg/ml, while the control group consisted of DMEM-HG supplemented with 10 % EV free FBS and 1 % penicillin-streptomycin with no added EVs, for 24 h. Cell migration was evaluated at the time points of 0, 6, 12, and 24 h by an inverted light microscopy. Three randomly selected fields in each well were used to calculate the scratch area using the ImageJ software.

### 2.10 Cell proliferation

HUVECs (endothelial cells) and HS68 (fibroblast cells) (Bioresource Collection & Research Center, Hsinchu, Taiwan) were incubated in EBM2 supplemented and DMEM-HG, supplemented, respectively. Endothelial cell medium was supplemented with a concentration of 50 μg/mL EVs derived from hASC cell monolayers, cell sheets, and cell spheroids. Fibroblast cells were supplemented with either a concentration of 25 μg/mL or 50 μg/mL EVs derived from hASC cell monolayers, cell sheets, and cell spheroids. In either type of cell, after 24 h had transpired, the medium was removed, and the cells were washed with PBS thrice, after which the Alamar Blue solution (AbD Serotec) diluted in the appropriate cell medium was directly added into the culture wells and the plate further incubated at 37 °C for 2 h. The fluorescence of experimental and control wells is read at Ex/Em 560/590 nm with a standard spectrophotometer (Tecan).

### 2.11 Tube formation assay

HUVECs were seeded into a 24-well plate coated with Matrigel (Corning, Corning, NY, USA) at a density of 5 x 10^4^ cells/well with special care to achieve a homogeneous distribution of the cell throughout the Matrigel film coating. These cells were cultured in a mixture of 90 % endothelial basal medium (EBM; PromoCell, Heidelberg, Germany) with 10 % EGM. Additionally, 50 μg/mL EVs from either monolayers, cell sheets, or hASC cell spheroids were added into the medium, respectively. A tube formation assay was performed as described [36]. EBM was used as a negative control, while EGM was used as a positive control. At 6 h, the formation of tube-like structures was visualized by a phase-contrast fluorescent microscope, and the images were analyzed using ImageJ.

### 2.12 *In vivo* wound healing experiments

The animal experiment was approved by the Institutional Animal Care and Use Committee of Taipei Medical University. Male Wistar Rats, 8–12 weeks old, were induced with diabetes using low-dose STZ (Streptozotocin) approximately 60 mg/kg. Rats were weighted, and their blood glucose level recorded daily to confirm diabetes, for a total period of fourteen days. After rats were anesthetized with pentobarbital sodium (30 mg/kg), they were randomly divided into four groups (n = 4) for EV addition, including the control, monolayer, cell sheet and cell spheroids. Treatments (i.e., monolayer EVs, cell sheet EV, cell spheroid EV or PBS devoid of EVs) were applied four times around the wound in a cross pattern on day 0 only, via topical application over the wound area covering the wound with chirurgical dressing. Each dose contained 50 μg of EVs or PBS without EVs for a total of 200 μg per wound site. Photographs of the wounds were taken on days 0, 2, 6, 10, and 14 and wound size was quantified by digital processing using ImageJ software. Wound size was calculated as the percentage of area of the wound versus the wound size on day 0.

### 2.13 Histology, immunohistochemistry and immunofluorescence analysis

Animals were sacrificed on postoperative day 14, and the damaged skins were excised and fixed overnight in 4 % paraformaldehyde. The fixed skins were then embedded in paraffin, cut into slices for H&E and Masson's staining according to universal protocols. For the immunohistochemistry and immunofluorescence analyses, the skin slices were rehydrated, incubated in citrate antigen retrieval solution, blocked with 1 % bovine serum albumin, stained using primary antibodies for CD31 (Anti mouse CD31 antibody, Service Bio, Wuhan, China). Slides then were incubated with appropriate secondary antibodies. The images of the slides were captured by Nikon Eclipse Ti2-U microscope and analyzed by ImageJ software.

### 2.14 Statistical analysis

All quantitative data were analyzed with GraphPad Prism 5.0 software (GraphPad Software Inc., San Diego, CA, USA), and shown as mean ± standard deviations (SD). The significances were analyzed by using the Student's t-test for two groups comparisons and one-way ANOVA with post hoc for multiple groups comparisons. Statistical significance was determined at P < 0.05.

## 3 Results

### 3.1 Selection of hASC spheroid size

**Fig. 1:**
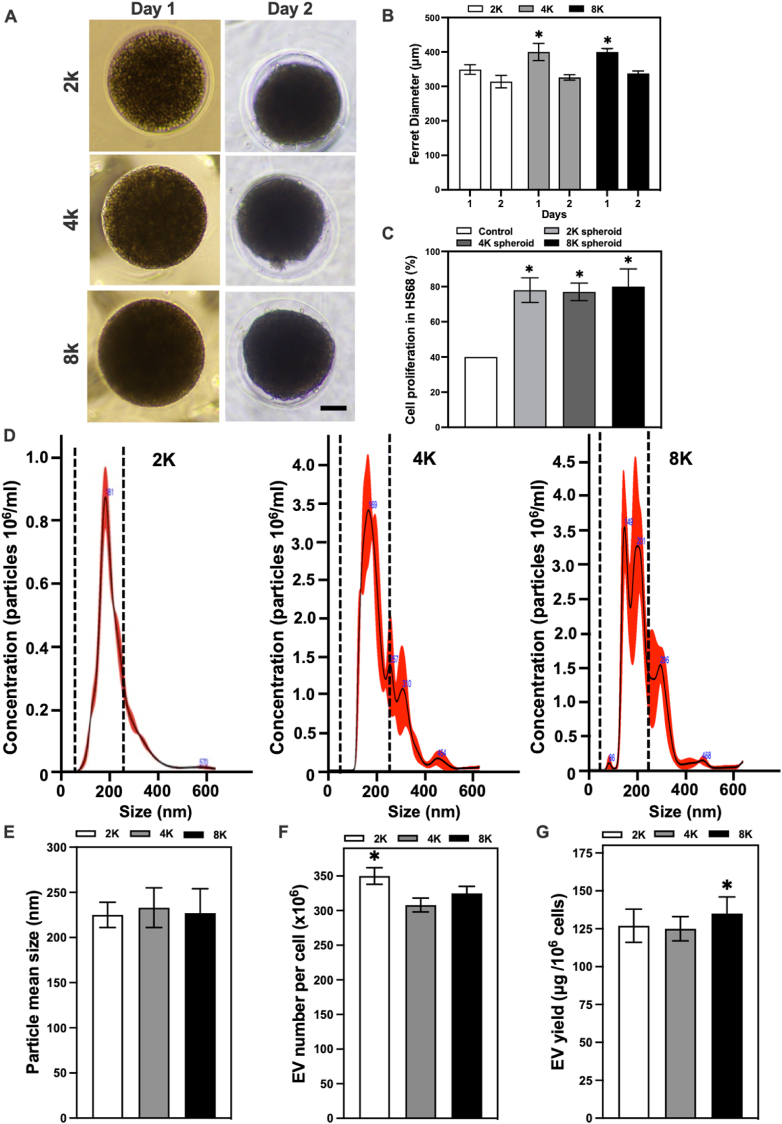
hASCs cultured in microwells at different seeding densities to form different-sized spheroids. **A** Representative microscopic images of hASC spheroids formed by respective cell number (2 K, 4 K, and 8 K; scale bar = 200 μm). **B** Ferret diameter of 2 K, 4 K, and 8 K spheroids was estimated at days 0 and 1 (n = 3; ∗*p* < 0.05 relative to the 2 K spheroid group). **C** Cell viability in HS68 fibroblast incubated with the EV groups at 50 μg/ml (n = 4). **D** hASC EV size distribution from 2 K, 4 K, and 8 K cell spheroid. **E** hASC EV mean size from 2 K, 4 K and 8 K cell spheroid. **F** hASC EV number per cell produced from 2 K, 4 K, and 8 K cell spheroid. **G** hASC EV yield per million cells from 2 K, 4 K, and 8 K cell spheroid. (∗P < 0.05, ∗∗P < 0.01, ∗∗∗P < 0.001, relative to the 2k spheroid hASC group).

With the aim to delimitate the conditions of fabrication for cell spheroids for future experiments and to select a series of fabrication conditions that effectively represent as broadly as possible the effects that the cells spheroid model has on the production of EVs and their effectiveness, modifications to the cell seeding density were carried out with different seeding densities started to form spheroids of various sizes in the microwells. During in vitro culture, the measured spheroid diameter decreased gradually in each group of ASC spheroids with different seeding densities (Fig. 1A). Ferret diameter for the hASC spheroids, initial cell densities of 2000 (2 K), 4000 (4 K), and 8000 (8 K) cells/per microwell were 349 m, 400 m, and 400 μm at the day of formation of the spheroids (day 1) respectively. The diameters of the spheroids were measured the following day (day 2) for a value of 314 m, 326 m, and 338 m, respectively. The diameters of the 4 K and 8 K groups were significantly larger than the 2 K group (Fig. 1B). Nanoparticle tracking analysis alongside BCA assay was employed, allowing for the precise quantification and characterization of particles at the nanoscale. Identification of the size, size distribution, concentration, and yield of the isolated EVs was possible (Fig. 1D–G). The results indicated that the vast majority of the EV produced by the spheroids groups had a size range lower than 200 nm, be these 2 K, 4 K, or 8 K (Fig. 1D). A comparison of the average size of the EV produced by the various spheroid groups did not indicate a significant difference in their size, with the average size of EV being 227 nm (Fig. 1E). On the other hand, an analysis of the number of EVs produced per cell number for each of the spheroids groups indicated that the 2 K cell spheroids were capable of producing more EVs, with a slightly higher value of EVs produced (350 EV per cell) when compared to the 4 K (308 EV per cell) and 8 K groups (325 EV per cell) (Fig. 1F). However, when comparing the yield of each of the spheroid groups via the amount of protein produced by each of them, 8 K spheroids were shown to produce slightly higher amounts of protein (135 μg) compared to 4 K (125 μg) and 2 K (127 μg) (Fig. 1G).

**Fig. 2:**
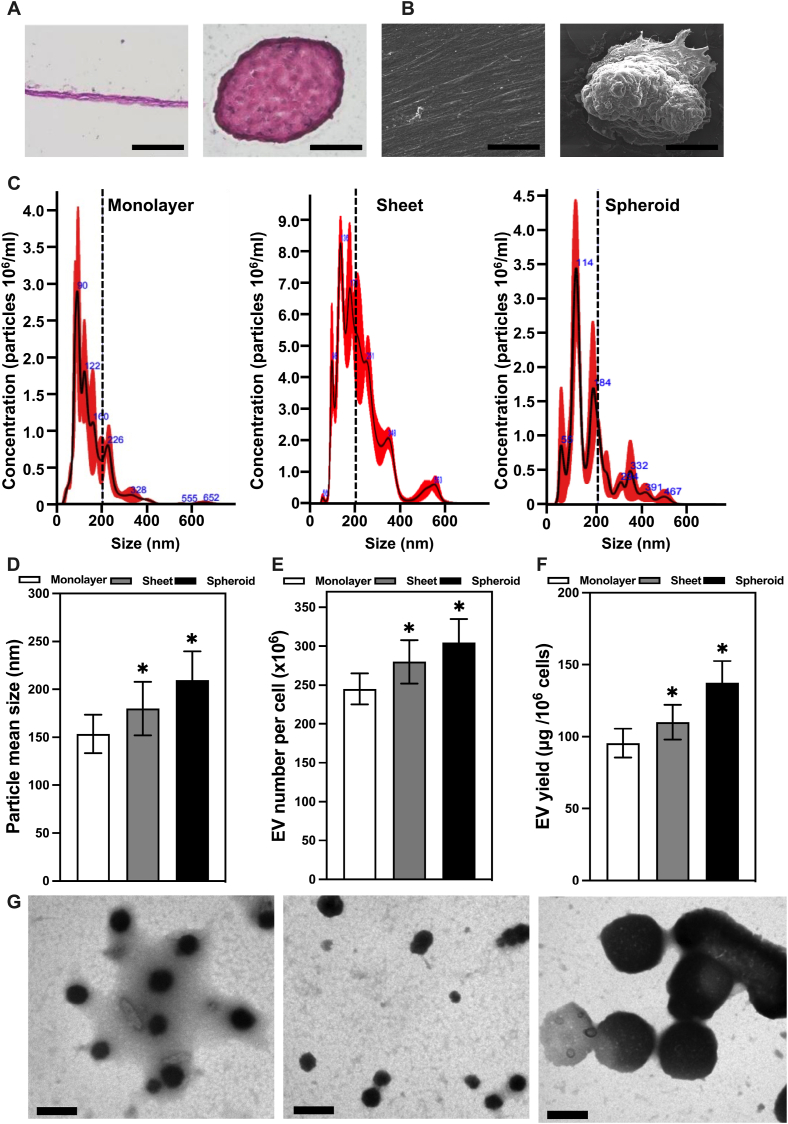
hASCs cultured in cell sheets and cell spheroids. **A** Representative H&E histological images of cell sheets and 4 K hASC spheroids (scale bar = 50 μm). **B** Scanning electron microscopic (SEM) images representative of hASC cell sheets and 4 K cell spheroids in top view (scale bar: 50 μm). **C** hASC EV size distribution from cell monolayer, cell sheet and cell spheroid. **D** hASC EV mean size from cell monolayer, cell sheet and cell spheroid. **E** hASC EV number per cell produced from cell monolayer, cell sheet and cell spheroid. **F** hASC EV yield per million cells from cell monolayer, cell sheet and cell spheroid. **G** Transmission electron microscope(TEM) images of EV obtained from cell monolayer, cell sheets and 4 K cell spheroids respectively (scale bar: 200 nm). (∗P < 0.05, ∗∗P < 0.01, ∗∗∗P < 0.001, relative to the monolayer hASC group).

An additional experiment was conducted to investigate the relation between the various EVs obtained from the spheroid groups (2 K, 4 K, and 8 K) and if there was any significant change in the angiogenic properties of these EVs via cell proliferation of HS68 cells. The results indicated no significant change in the proliferation of the cells when exposed to the different EVs derived from the spheroid groups (Fig. 2C). Considering these results and the information shown in the relevant literature [37], which indicates that the oxygen tension gradient differs by less than 10 % from the outer surface of even the largest MSC spheroids up to 350 μm in diameter. Together with our previous study [32] that has confirmed the increase of angiogenic growth factors on angiogenesis-related genes on 8 K spheroids, the following experiments were carried out using 4 K spheroids.

### 3.2 hASC characterization

In order to confirm the successful formation of the morphologies desired for the production of EVs, a series of characterization tests were carried out to identify the sheet and spheroid morphologies. These were achieved through the histological examination of both cell sheets and cell spheroids by H&E staining. Through these, it was revealed that the structure of hASC sheets consisted of an average of two to three layers of cells free from gaps, tightly arranged, and packed in a considerably small area, presenting a robust flat structure. Meanwhile, the hASC spheroids, in particular the innermost center of the spheroid structure, exhibited a highly compact formation of cellular aggregates with distinctive ECM formation. This structural differentiation between the inner and outer layers in the spheroid structure was readily observed due to the discoloration observed closer to the center of the spheroid (Fig. 2A).

To deepen our understanding of the structural differences of these morphologies, scanning electronic microscopy (SEM) was carried out to obtain a more detailed picture of the external structure of hASC sheets and spheroids. For the cell sheet, through the SEM analysis, it was observed that the outermost layer of cells exhibited a quasi-smooth surface. In relation to this unique characteristic, it was also observed that the alignment of these cells was uniform in regard to their direction (Fig. 2B). As a result of these two characteristics, the surface of the cell sheet displayed a remarkable degree of uniformity, lacking any discernible cell boundaries. Further analysis of the images indicated the presence of a robust and well-developed matrix within the cell sheet. The matrix, in this context, refers to the extracellular substances secreted by the cells, such as proteins and polysaccharides, which play a vital role in cell adhesion and tissue structure. The abundance of matrix formation on the surface of the cell sheet contributed to its overall smooth appearance, as it concealed any clear demarcation between individual cells. In contrast to the quasi-smooth and aligned surface of cell sheets observed before, the SEM analysis of hASC spheroids revealed a distinct topographical difference, with the surface of hASC spheroids exhibiting a markedly uneven or irregular appearance. This unevenness stemmed from the behavior of cells on the spheroid's top surface, where they appeared to aggregate closely together. Unlike the cell sheet, on which cells aligned uniformly, the cells on the top surface of the spheroids exhibited a tendency to cluster tightly, forming denser cell aggregates. Such clustering of cells on the spheroid surface was easily noticeable in the SEM images, making it challenging to differentiate individual cell boundaries or delineate clear boundaries between adjacent cells. Such variations in surface topography between cell sheets and spheroids highlighted the unique structural characteristics associated with these two different cell culture models. While cell sheets presented a smoother, matrix-rich, and more aligned surface, spheroids, in this case, showcase a rougher and densely clustered top surface, making it challenging to identify individual cell boundaries. Instances of phenomena like these have already been documented in a variety of different 3D spheroid models in which an even greater variety of cells have been used for an even greater variety of reasons ranging from tumor models to studies of the effects the changes in the extracellular matrix can have in the overall behavior of the cells [32,[38], [39], [40]]. As such, significant advantages like increased ECM production, composition, and organization offered by the spheroids cell model over traditional cell culture methods and cell sheets (2D) are of great use. The combination of these well-established characteristics and advantages with our model is sure to play a significant role in the production of EVs obtained from these spheroids.

Analysis of the surface markers of hASCs with flow cytometry was also carried out (Fig. S1). The results of this analysis revealed a distinct pattern of surface marker expression, shedding light on the unique identity of these cells. Notably, the analysis indicated that hASCs exhibited a negative expression for CD31 and CD34, while displaying positive expression for CD44, CD73, CD90, and CD166. These results are significant as they align with the characteristic surface marker expression pattern that is commonly associated with mesenchymal stem cells (MSCs), to which hASCs belong. MSCs are known for their distinctive surface marker profile, which typically lacks the expression of endothelial markers such as CD31 and CD34, while concurrently exhibiting positivity for markers such as CD44, CD73, CD90, and CD166. These markers are indicative of the mesenchymal lineage and are frequently utilized as defining criteria for identifying and characterizing MSCs. The conformity of hASC surface marker expression to the established MSC profile underscores their identity as a subset of mesenchymal stem cells.

### 3.3 Physical characterization of EVs

Extracellular vesicles (EVs) isolated from the different hASC cell cultures via size exclusion chromatography were analyzed. To this end, nanoparticle tracking analysis (NTA) was used. This sophisticated technique enabled the precise quantification and characterization of particles at the nanoscale. Both the size and the size distribution of the isolated EVs were thus identified. The results revealed a range of EV sizes across the different experimental groups, spanning from approximately 50 to 220 nm (Fig. 2C). Of particular importance, a shift in the most prevalent peaks of sizes of the EVs between the monolayer and spheroid groups was observed, with the main-sized peaks in the monolayer group being 90 nm and 120 nm, while those in the spheroid group consisted of 114 nm and 184 nm. 10.13039/100014337Furthermore, the analysis of the mean EV size obtained from this technique supported the findings alluded to in the size distribution analysis as cell monolayer, cell sheet, and cell spheroid EVs exhibited a mean size of 153.5 ± 27 nm, 180 ± 30 nm, and 209 ± 38 nm, respectively (∗P < 0.05 relative to the monolayer hASC group) (Fig. 2D). After the measurement of the size and size range of the hASC EVs was determined via NTA, this same technique was used to ascertain the concentration of EVs produced by the various hASC groups. Increases in concentration were detected and recorded with a more pronounced increase in the cell spheroid group with a value of 300 EV per cell, followed by those of cell sheet with a value of 280 EV per cell, and lastly, cell monolayer with a value of 245 EV per cell (Fig. 2E). These results indicated a significant boost in the production of EVs after the cells were subjected to a change in the morphology of their culture methods. This information is of paramount importance, as it provides critical insights into the heterogeneity of the EV population within each group as well as the increase presented in the number of EVs produced. This analysis and the variability observed in the size distribution are consistent with the inherent variability often encountered in extracellular vesicle populations, as EVs are known for their diverse cargo and functional roles, which can vary depending on their size and content. At the same time, it provides valuable evidence of the enhancement that the different cell culture morphologies have on the production capacity of EVs.

Moving on from these analyses, the yields of the different EV groups were quantitatively assessed via BCA protein assay, a highly sensitive and widely used method for quantifying protein content, which is a reliable indicator of EV production. This assay enabled us to precisely measure the amount of protein within each EV group and, consequently, calculate the EV yield per million cells. Notably, hASC cell spheroids demonstrated the highest yield, with an impressive production rate of 137.5 ± 15 μg of EVs per million cells. Following closely, hASC cell sheets exhibited a substantial EV yield, amounting to 110.1 ± 12 μg per million cells, while hASC cell monolayers, still yielding a considerable 90 μg of EVs per million cells, demonstrated a slightly lower production rate compared to the other two groups. (∗P < 0.05, relative to the monolayer hASC group) (Fig. 2F).

A further analysis of the previously described quantitative results was carried out via SEM imaging of the different EVs to corroborate the size difference registered in the NTA analysis. From the images obtained, it was clear that a difference in the sizes of the EV could be correlated with the morphology of the cells that produced them. The data regarding the size of the EV was recorded as 127 nm for monolayer, 183 nm for sheet, and 261 nm for spheroids (Fig. 2G).

These results reaffirm the exceptional capacity of cell spheroids to generate EVs while providing valuable comparative data that emphasizes the efficiency of cell spheroids in generating EVs when compared to cell sheets and monolayers. By combining the results of NTA as well as the BCA assay, we have established a comprehensive characterization of the isolated EVs. This multifaceted approach not only confirms their identity as EVs, but also provides valuable information about their size distribution and diversity.

### 3.4 Protein and uptake characterization of EVs

**Fig. 3:**
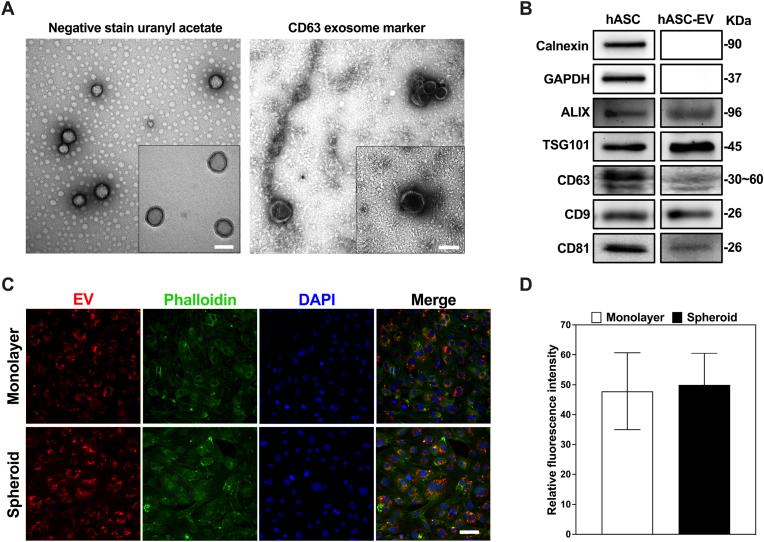
Protein characterization of extracellular vesicles and cell uptake. **A** Representative transmitting electron microscopic images of hASC EVs with uranyl acetate and CD63 labelling (scale bar: 100 nm). (n = 3). **B** Expression of the EV-related markers, Calnexin, GAPDH, ALIX, TSG101, CD63, CD9 and CD81 in EV-monolayer as determined through western blotting. **C** Confocal images of HUVEC cells exposed to monolayer or spheroid EVs. Red: EVs labeled with Cy5. Green: Cell cytoskeleton stained with phalloidin Green. Blue: nuclei stained with DAPI. (scale bar: 50 μm). **D** Fluorescence intensity quantitative analysis. (∗P < 0.05, ∗∗P < 0.01, ∗∗∗P < 0.001, relative to the monolayer hASC group).

Following the physical characterization of the various groups of EVs, a further analysis of the EVs obtained from these was carried out. To this end, a crucial step in characterizing these EVs was employed by transmission electron microscopy (TEM). This powerful imaging tool enabled us to visualize the morphology and structural features of the isolated vesicles. Furthermore, to enhance our understanding of the specific subtype of EVs within the isolated population, we carried out parallel experiments involving the labeling of the EV surface with CD63 (Fig. 3A). As a well-established marker, CD63 is commonly associated with EVs, a subcategory of EVs with distinct biogenesis and functional characteristics. The CD63 labeling results were particularly illuminating, as they indicated that a substantial proportion of the isolated extracellular vesicles indeed exhibited a positive CD63 surface marker expression. This finding strongly suggests that a significant portion of the EVs isolated from hASC cell spheroids can be classified as EVs. Therefore, the combination of size exclusion chromatography, TEM visualization, and CD63 labeling not only validated the successful isolation of EVs from hASC spheroids but also allowed us to identify a substantial subset of these EVs as EVs confidently.

Most EVs have conserved a set of proteins, such as heat shock proteins and certain members of the tetraspanin superfamily of proteins, especially CD9, CD63, CD81, and TSG101. We demonstrated the expression of these markers in hASC EVs by western blot and identified EV-specific proteins in hASC EV samples, including ALIX, TSG101, CD61, CD9, and CD81 (Fig. 3B).

Fluorescence staining was utilized to visualize and track the interaction between EVs obtained from hASC monolayer and spheroids and HUVEC cells in vitro. A visual examination of the internalization process of EVs obtained from hASC monolayer and spheroids was conducted (Fig. 3C–D). Through these observations, a series of fascinating insights were ascertained. First of these was that both groups of EVs, whether derived from hASC monolayer or spheroids, exhibited a remarkable capacity for internalization and accumulation by HUVEC cells. This was demonstrated by the intensity of the fluorescence signal associated with the EVs present within the cellular environment and its cytoskeleton observed in the imaging. A subsequent insight upon closer analysis of the images, centered around the fluorescence intensity of the red-dyed EVs, indicated that no significant difference was found in the uptake rates between the two groups. This discovery suggests that both EV groups possess a similar ability to both reach and be assimilated into the target cells.

### 3.5 miRNA data analysis

**Fig. 4:**
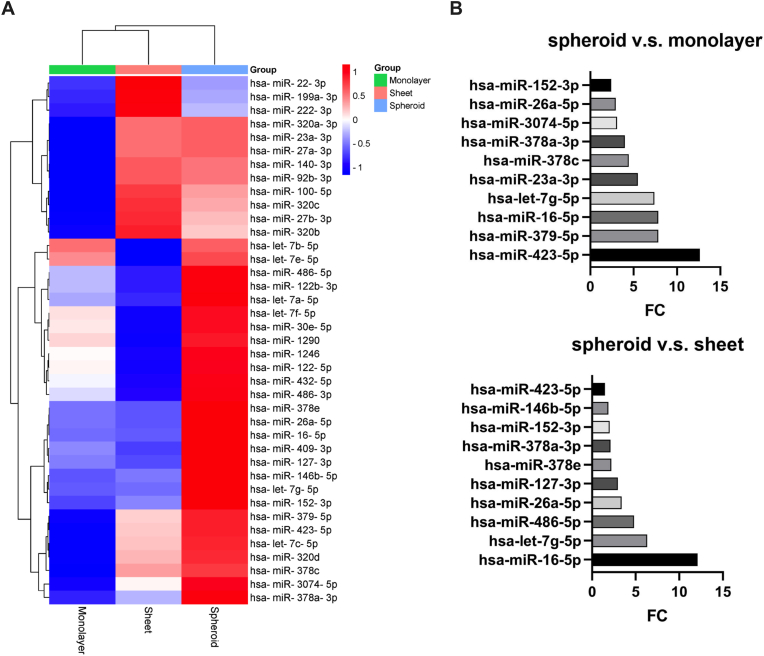
miRNA analysis of extracellular vesicles **A** Heatmap displaying specific names and expression levels of differentially expressed miRNAs in monolayer, sheet and spheroid. **B** Bar graph summarizing the 10 main specific differences in differentially upregulated miRNAs in the spheroid group.

Following the characterization of the various groups of EVs, changes in the miRNA contained within the EVs were similarly analyzed in order to understand what changes these had suffered and the relation of these changes with the different EV groups. Therefore, a miRNA-seq assay was performed to determine the differences between spheroid, sheet, and monolayer EVs. Compared to monolayer-derived EVs, any and all twofold changes in the micro-RNA were identified in spheroid and sheet-derived EVs. A total of fourteen miRNAs overlapped between the groups were identified, while 59 miRNAs were found in the sheet EV group, and 44 miRNAs were identified in the spheroid EV group. EVs derived from 3D cultures of hASCs exhibited superior efficacy compared to those from 2D cultures. We propose that 3D culturing alters the EV cargo composition, with unique miRNAs in EVs from 3D-cultured hPMSCs contributing to enhanced immunomodulatory and regenerative properties. To investigate this, we performed miRNA microarray analysis to assess the miRNA expression profiles of EVs from 3D spheroid, cell sheet, and 2D monolayer cultures of hASCs. After probe screening and data normalization, we identified 39 significantly differentially expressed miRNAs in EVs from 3D cultures compared to cell sheet and 2D monolayer cultures (Fig. 4A). We then quantitatively compared miRNA expression levels across these conditions, focusing on the ten miRNAs with the highest fold changes. Notably, miR-16-5p, miR-7g-5p, miR-26a-5p, miR-378a-3p, miR-152-3p, and miR-423-5p were markedly upregulated in EVs from 3D cultures. The heatmap and bar graphs together provide a comprehensive overview of miRNA expression variations across the different culture conditions. These findings were further validated by RT-PCR (Fig. 4B).

**Fig. 5:**
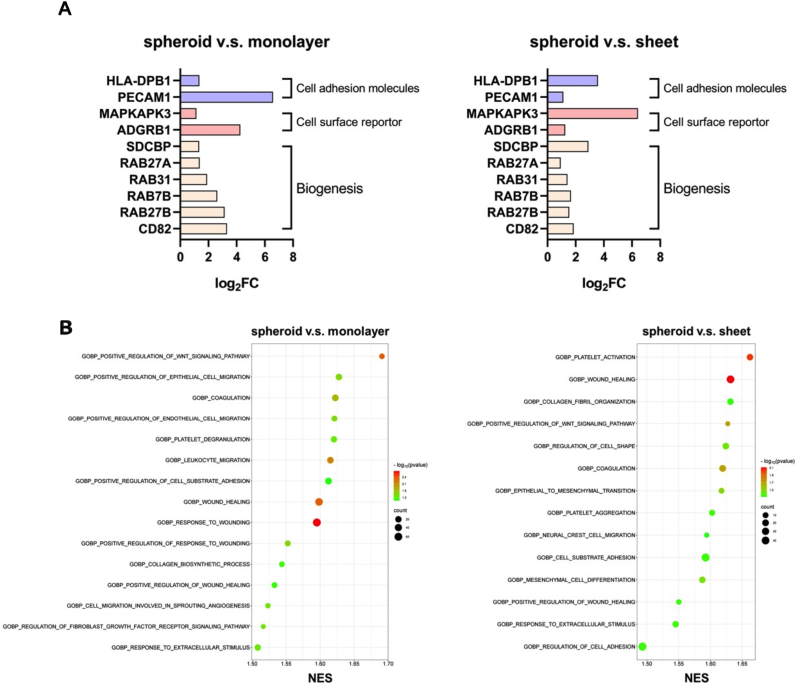
RNA sequencing and enrichment pathways. **A**n Upregulated genes fold change involved in EV biogenesis, cell surface reporter, and cell adhesion pathways determined by RNA-sec. **B** Bubble charts enrichment pathway involvement of differently expressed genes based on RNA-seq.

We performed RNA-seq analysis of EVs derived from 3D spheroid, cell sheet, and 2D monolayer cultures to identify potential mechanisms underlying the promoted EV secretion. Differentially expressed genes (DEGs) with a log2 fold-change >1.0 and a false discovery rate (FDR) < 0.05 were identified and analyzed to assess transcriptomic differences between samples. Recent studies have highlighted the critical role of EV functions in promoting wound repair, including mediators such as cell adhesion molecules, cell surface receptors, and biogenesis-related genes. In our study, EVs derived from hASC spheroids showed significant upregulation of biogenesis-related genes (SDCBP, RAB27A, RAB31, RAB7BA, RAB27B, and CD82) compared to those from cell sheet and 2D monolayer cultures, indicating that 3D cellular organization enhances biogenesis (Fig. 5A). Additionally, EVs from 3D spheroid cultures exhibited enhanced cell adhesion and cell surface transport functions, as evidenced by the upregulation of cell adhesion genes (HLA-DPB1 and PECAM1) and cell surface reporter genes (MAPKAPK3 and ADGRB1). Gene ontology (GO) analysis further revealed that EVs from 3D spheroids had enriched gene signatures related to the positive regulation of the WNT signaling pathway, epithelial and endothelial cell migration, coagulation, wound healing, and collagen fibril organization (Fig. 5B).

### 3.6 Scratch assay

**Fig. 6:**
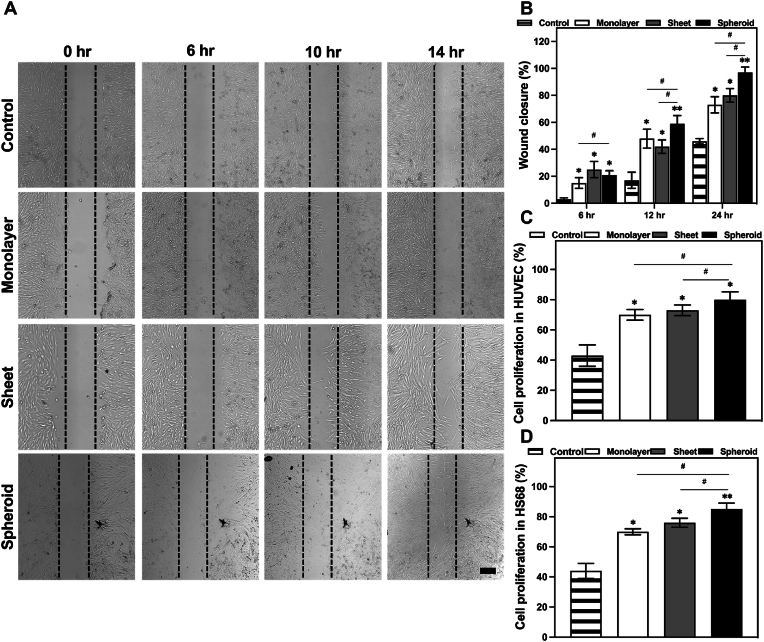
EVs enhanced HS68 fibroblast cell migration in vitro. **A** Cell scratch assay as observed under a light microscope at different time periods (scale bar: 500 μm) **B** The scratch closure rate analysis presented over time (n = 3) **C** Cell viability in HUVECs incubated with the different EV groups at a 50 μg/ml (n = 4). **D** Cell viability in HS68 fibroblast incubated with the EV groups at different concentrations (n = 4). (∗P < 0.05, ∗∗P < 0.01, ∗∗∗P < 0.001, relative to the control group). (#P < 0.05, ##P < 0.01, ###P < 0.001, relative to the monolayer group).

To further understand the impact the various EV groups (derived from hASC monolayer, cell sheets, and cell spheroids) possess, specifically on the cell migration abilities of HS68 fibroblast cells, a series of scratch assays were conducted, using uniform EV concentrations across all groups, in order to assess the changes in cell migration. This experimental approach allowed an in-depth exploration of the potential effects of these EV groups on the wound healing ability of the fibroblast cells (Fig. 6A). The results of these scratch assays yielded important findings. One of the most noteworthy was how all EV groups displayed a remarkable capacity to significantly accelerate the migration of HS68 fibroblast cells when compared to the control group (serum-free medium). This capacity to significantly accelerate cell migration underscores the regulatory role of EVs in promoting cellular motility and wound closure.

Further analysis of the data revealed significant variations in the effectiveness of the EV groups in enhancing cell migration. Foremost of them all, the cell spheroid EV group demonstrated the most robust and consistent performance, as it not only induced an accelerated cell migration at various time points, but also maintained its effectiveness throughout the 24-h assessment period. Following closely in effectiveness, the cell sheet EV group also exhibited a substantial impact on cell migration, contributing to a faster closure of the wound gap. Lastly, the cell monolayer EV group, while still promoting migration, displayed a slightly lower enhancement compared to the other two groups. A quantitative analysis of these results clearly illustrated the varying degrees of influence (Fig. 6B): the cell spheroid EV group achieved an impressive 97 % ± 4 % total percentage of cell migration, followed by the cell sheet group with an 80 % ± 5 %, and the cell monolayer group with a still substantial 73 % ± 6 % enhancement. These values clearly indicate the impact of EVs in promoting wound healing and cellular migration, compared to the control group (serum-free medium 46 % ± 2 %)

### 3.7 Cell proliferation

To further evaluate the effects the different EV groups have in the wound healing process, a comprehensive assessment was carried out regarding the impact of the various EV groups on the critical aspects of wound healing, particularly focusing on their ability to enhance cell proliferation. We conducted this assessment using two distinct cell lines, HUVEC endothelial cells, and HS68 fibroblast cells, both of which play essential roles in the wound healing process.

For our first set of experiments, the Alamar Blue assay was used to evaluate the effects of EVs from different groups on HUVEC cells at a fixed concentration. The results of this assay indicated a significant increase in HUVEC cell viability across all EV groups when compared to the control group treated with phosphate-buffered saline (PBS). This notable enhancement of cell viability provided compelling evidence of the positive influence EVs exert on the proliferative potential of HUVEC cells. Furthermore, the data obtained clearly indicated a group-dependent response, with the EV group derived from cell spheroids leading the way with a remarkable 80 % ± 5 % cell viability. Following closely, the cell sheet group exhibited a substantial 73 % ± 4 %, and the cell monolayer group achieved a notable 70 % ± 4 % cell viability, all in contrast to the control group's 43 % ± 7 % viability (Fig. 6C).

In parallel, a second set of experiments was conducted using HS68 fibroblast cells and varying concentrations of EVs. The results validated that the proliferation effect of EVs indeed follows a dose-dependent pattern. When tested at a concentration of 25 μg/mL, the various EV groups displayed a lower, yet still significant, impact on cell proliferation. The cell monolayer group resulted in 62 % ± 3 % cell viability, the cell sheet group in 69 % ± 2 %, and the cell spheroid group in a notable 72 % ± 2 % cell viability (Fig. 6D).

When comparing these results with those achieved when a higher concentration of EVs was used (50 μg/mL), the difference in treatment efficacy was apparent. At this higher concentration, the proliferation effect was substantially amplified. The cell monolayer group showed a remarkable 70 % ± 2 % of cell viability, the cell sheet group an impressive 76 % ± 3 %, and the cell spheroid group demonstrated the highest level of proliferation with an outstanding 85 % ± 4 % level in cell viability. These findings underscore the substantial potential of EVs from different sources in promoting cell proliferation, with the effects being not only statistically significant but also dose-dependent. The concentration-dependent response further emphasizes the capability of EVs to enhance wound healing by accelerating cell proliferation.

### 3.8 Tube formation assay

An additional in-depth analysis of the angiogenic properties of distinct EV groups was carried out employing a tube formation assay with HUVEC cells as our chosen model. Due to their stem cell-like potential and their ability to form intricate tubular structures within the extracellular matrix, closely mimicking the process of blood vessel formation, these characteristics make them ideal for investigating the impact of EVs on the tube formation capability of cells, a pivotal aspect of angiogenesis.

The results of this assay unveiled the significant angiogenic potential of all EV-exposed groups. Specifically, a substantial increase in the presence and number of tubes formed by HUVECs was observed, indicative of their enhanced angiogenic behavior.

**Fig. 7:**
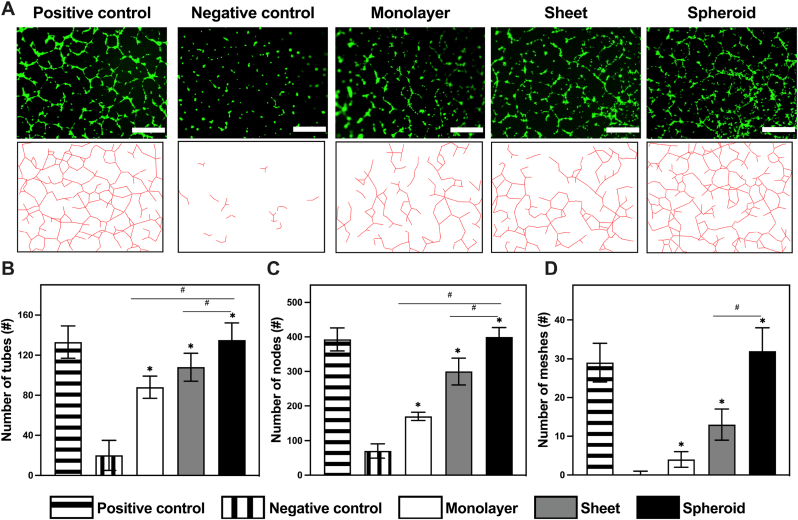
Fluorescence microscopy imaging and quantification of tube formation. **A** Fluorescent microscopic images of tubes formed by HUVECs in the tube formation assay (scale bar: 500 μm). **B** ImageJ analysis of the total meshes. **C** ImageJ analysis of the total nodes. **D** ImageJ analysis of the total tubes. (n = 4). (∗P < 0.05, ∗∗P < 0.01, ∗∗∗P < 0.001, relative to the control group). (#P < 0.05, ##P < 0.01, ###P < 0.001, relative to the monolayer group).

HUVECs treated with EVs derived from the various hASC culture groups formed compact, well-structured, tube-like networks, closely resembling the patterns observed in the positive control group (Fig. 7A). In stark contrast, the negative control group exhibited a disrupted network with numerous discontinuous branches and non-connected nodes, underscoring the vital role of EVs in promoting angiogenesis. Among the EV groups, the spheroid EV group stood out with its extraordinary performance, significantly surpassing both the control group and the other EV groups in terms of tube formation.

To quantitatively evaluate the tube formation of HUVECs, we employed ImageJ to analyze cell images and extract valuable data. This analysis allowed us to measure the total number of meshes, nodes, and segments per visual field, which were then compared to the negative control group for reference (Fig. 7B–D). Across all the parameters analyzed, including the number of meshes, nodes, and segments, the spheroid EV group exhibited a highly significant improvement, approaching values that closely resembled those of the positive control group. Respectively, the values for each of these parameters were: 32 ± 6 for meshes, 400 ± 27 for nodes, and 135 ± 17 for extremities.

Following the spheroid EV group in terms of their angiogenic impact were the cell sheet with values of 13 ± 4 for meshes, 300 ± 39 for nodes, and 108 ± 14 for extremities. Finally, the monolayer EV groups presented values of 13 ± 4 for meshes, 300 ± 39 for nodes, and 108 ± 14 for extremities. Both of these groups demonstrated a notable enhancement in the angiogenic capabilities of HUVEC cells, as evidenced by their increased tube formation. However, it is worth noting that the formation of meshes in these groups was not as pronounced due to the presence of numerous non-connecting nodes and isolated segments.

### 3.9 *In vivo* wound healing experiments

In the progression of our study, a critical step was taken to examine the angiogenic potential of distinct EV groups in an in vivo setting, specifically in a diabetic wound healing model. To achieve this, a previously established wound model in diabetic rats was used, and then carefully monitored for the effects (wound area, contraction, infection, etc.) of these EVs over a period of fourteen days. This specific timeframe was chosen thoughtfully, as it aligned with the known duration of skin wound healing in rats, typically spanning two weeks. As such, it serves as a pertinent short-term model to study cutaneous wound healing processes pertinent to the effects of EVs. Throughout this timeline, the healing progress was observed alongside the general health of the experimental subjects, which included regular measurements of body weights and blood glucose levels.

**Fig. 8:**
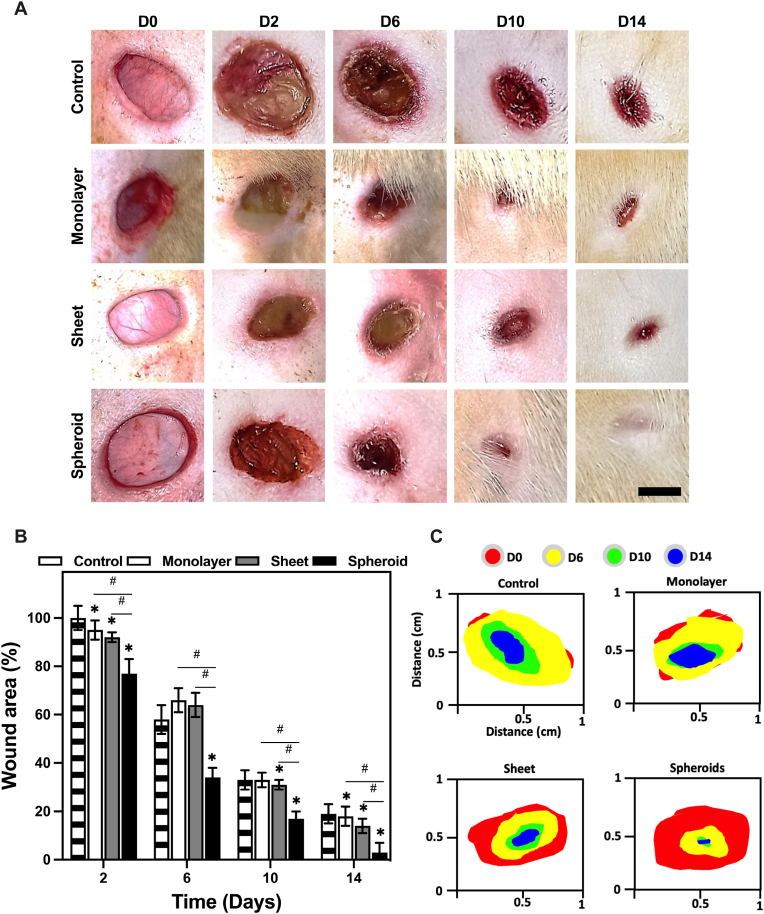
EV effects on diabetic rat puncture wound healing model. **A** Representative images of rat wounds over the length of the experiment. **B** Closure evaluated for wounds treated with the different EV groups. **C** The wound area at days 2, 6, 10, and 14 quantified by ImageJ software. (n = 4). (∗P < 0.05, ∗∗P < 0.01, ∗∗∗P < 0.001, relative to the control group). (#P < 0.05, ##P < 0.01, ###P < 0.001, relative to the monolayer group).

All the diabetic rats in the study presented body weights exceeding 40 g and elevated blood glucose levels. It is important to note that no significant differences were observed either in the body weight or blood glucose levels among the different EV treatment groups, indicating that the health status of the animals remained stable throughout the experimental period. To monitor the progression of the different wound healing sites, these were diligently photographed at various time points over the fourteen-day observation period, providing a visual record of the healing process (Fig. 8A).

After consolidating the measurements from the various photographs of the distinct EV groups into a wound boundary graph (Fig. 8B), a comprehensive view of the efficacy of different EV groups in facilitating the healing process was quickly identified. From this, the reduction of the wound area could be readily observed over the fourteen-day period, allowing for a clear comparison of the healing rates among the control group and the EV-treated groups. Notably, the spheroid EV group consistently outperformed the other groups, with a remarkable and significantly faster closure rate. This is particularly evident in the wound boundary graph, where the spheroid EV-treated wounds showed a rapid decrease in wound size, reaching near-complete closure by day 14. In contrast, the control group exhibited a slower rate of wound closure, highlighting the substantial impact of EV treatment. The monolayer and cell sheet EV groups also displayed improved wound healing rates, further underscoring the potential of EVs in expediting the recovery process.

To quantitatively assess the wound closure rate as well as the area, the percentage of wound size relative to its initial size at different time intervals was analyzed. In the control group treated with PBS, wound closure progressed as follows: at day 2, it was approximately 58 % ± 6 %; at day 6, 33 % ± 4 %; at day 10, 19 % ± 4 %; and at day 14, it reached near-complete closure at 100 % ± 5 %. In contrast, the various EV-treated groups exhibited markedly different outcomes. For the monolayer EV group, wound closure proceeded as follows: on day 2, approximately 95 % ± 4 %; on day 6, 66 % ± 5 %; on day 10, 33 % ± 3 %; and on day 14, nearly complete closure at 18 % ± 4 %. Similarly, the cell sheet EV group demonstrated accelerated wound closure with values of approximately 92 % ± 2 % on day 2, 64 % ± 5 % on day 6, 31 % ± 2 % on day 10, and 14 % ± 3 % on day 14. Remarkably, the cell spheroid EV group outperformed all other groups, showing rapid wound closure rates of 77 % ± 6 % on day 2, 34 % ± 4 % on day 6, 17 % ± 3 % on day 10, and 3 % ± 4 % on day 14.

These results clearly indicate a significant increase in the wound closure rate in rats treated with EVs from the spheroid group, compared to those in the control group, at different time points following the induction of the wounds (Fig. 8C). It is noteworthy that, throughout the healing process of the puncture wounds, no signs of infection, necrosis, or noticeable wound contraction were observed in any of the EV treatment groups. Statistical analysis of the wound area reinforced the observation that, across all EV treatment groups, wounds consistently healed at a faster rate compared to the control group.

### 3.10 Histology, immunohistochemistry and immunofluorescence analysis

**Fig. 9:**
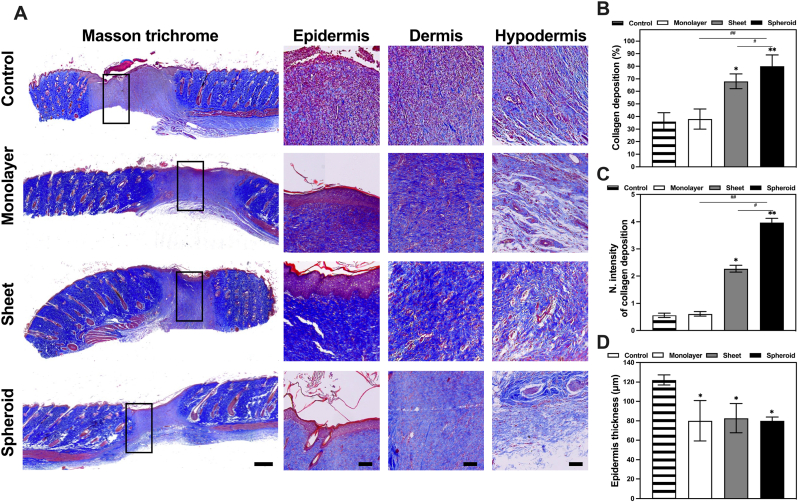
EV promoted granulation tissue regeneration, re-epithelialization, collagen formation, and remodeling during rat diabetic wound healing. **A** Masson staining in the skin wound sections (scale bar: 1000 μm and 100 μm). **B** IHC toolbox was used to semiquantify the blue collagen in Masson trichrome staining. **C** Normalized quantitative analysis of collagen deposition by ImageJ software. **D** The epidermal thickness was analyzed by ImageJ software. (n = 4). (∗P < 0.05, ∗∗P < 0.01, ∗∗∗P < 0.001, relative to the control group). (#P < 0.05, ##P < 0.01, ###P < 0.001, relative to the monolayer group).

At the fourteen days following the initial skin puncturing, the rats in all groups were anesthetized, the wound areas cut, and paraffin sections were prepared to assess the wound healing process. The skin regions of all groups were analyzed via H&E staining, Masson staining, and immunohistochemistry of CD31. The resulting images were semiquantitatively analyzed via ImageJ software (Fig. 9).

Firstly, the Masson trichrome staining provided a detailed histological analysis of the tissue samples to assess and visualize the various components and structures involved in the wound healing process, particularly the collagen regeneration and deposition on the skin samples. This analysis particularly shed light on the impact of EV treatments in the diabetic rat wound healing experiment (Fig. 9A). The results unveiled substantial structural changes and a remarkable augmentation of collagen deposition in the skin samples when compared to the control group. In an observable visual contrast to the control group, the EV-treated groups exhibited a significant increase in the regularity and arrangement of their collagen fibers. With significant reductions to the presence of anomalous, splintered, and haphazard collagen formations, replaced by organized and denser collagen networks. Furthermore, a discernible decrease in the presence of inflammatory cells was observed in the skin samples of the EV-treated groups compared to the control. This reduction in inflammation marked a significant step towards healthier tissue regeneration. Among all the treatments, it was the spheroid EV group that presented the highest increase of collagen deposition. This EV group showcased the most copious and densely packed collagen fibers, a highly compact stratum corneum, and an epidermal layer of comparable thickness to the other EV groups, indicating the remarkable potential of spheroid EVs in promoting wound healing.

For a more quantitative assessment of the collagen deposition in the tissue samples (Fig. 9B), imaging software (ImageJ) was utilized to perform semiquantitative analysis. The results were as follows: the control group displayed 36 % ± 7 % of collagen deposition; the cell monolayer group had 38 % ± 8 %; the cell sheet group demonstrated 68 % ± 6 %; and the cell spheroid group exhibited 80 % ± 9 % of collagen deposition. To delve even deeper into the understanding of collagen deposition, an analysis of the normalized intensity of collagen deposition (Fig. 9C) was carried out. This analysis provided a more comprehensive insight into the accumulation of collagen over a specific area. The results derived from this analysis indicated that the control samples presented significantly lower levels of collagen accumulation compared to the various EV groups. In ascending order of collagen accumulation, the groups were ranked as follows: the control group (0.56 ± 0.08), cell monolayer group (0.61 ± 0.08), cell sheet group (2.27 ± 0.13), and the cell spheroid group, which displayed the highest levels of collagen accumulation at 3.97 ± 0.15.

An additional study conducted involved the examination of the epidermis in the tissue samples (Fig. 9D) to observe the re-epithelialization properties of each EV group. Contrary to our previous findings, the control group exhibited a significantly higher epidermal thickness, with an average of 122 ± 4 μm. In contrast, the various EV-treated groups consistently presented significantly lower epidermis thickness levels, averaging around 80 ± 15 μm. This marked difference between the thickness of the control group and the different exosomes groups is likely an indication of the immature state of the wound healing process on the control group, as an increase in cell proliferation of keratinocytes (predominant cell type found in the epidermis) is one of the hallmarks of the early stages of the wound healing process. Such increased thickness is therefore not present in the exosome groups, which have already overcome this stage of the wound healing process.

Following these result, skin samples were further analyzed via CD31 immunofluorescence staining and H&E staining. The combination of these techniques offered a comprehensive evaluation of various critical processes, including angiogenesis and tissue structure regeneration.

**Fig. 10:**
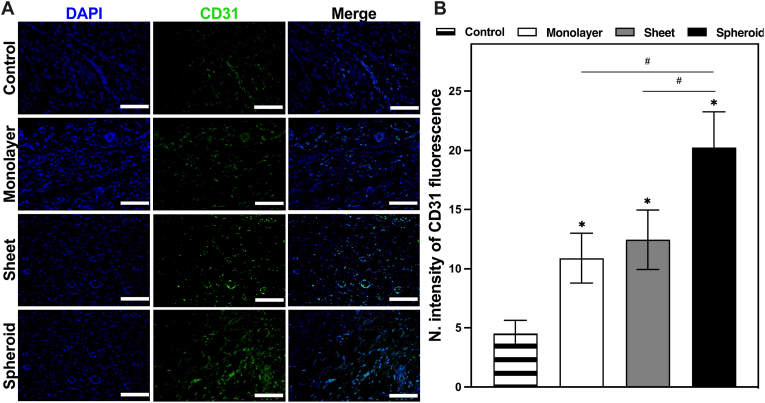
Animal tissue analysis for angiogenesis. **A** The immunofluorescence images of rat wound tissue. Green: CD31 staining with CD31 antibody. Blue: nuclei stained with DAPI **B** Semiquantitative immunofluorescence of wound sections. IHC toolbox was used to semiquantify the CD31 in immunofluorescence images. (∗P < 0.05, ∗∗P < 0.01, ∗∗∗P < 0.001, relative to the control group). (#P < 0.05, ##P < 0.01, ###P < 0.001, relative to the monolayer group).

CD31 immunofluorescence staining played a pivotal role in the investigation, enabling an in-depth exploration of the regenerative processes unfolding within the tissue samples. As CD31 is a platelet endothelial cell adhesion molecule, it serves as a valuable marker for identifying and quantifying new blood vessels. An essential aspect of angiogenesis. Through this staining, the presence of CD31^+^ vessel structures was easily visualized. In addition to this, analysis of their concentration and distribution within the tissue samples was smoothly identified (Fig. 10A). The fluorescent images obtained indicated the effectiveness of the aforementioned angiogenic abilities and the extent to which the various EV groups exhibited this particular trait. The differences in CD31 fluorescence signal were visibly apparent, with rats treated with cell EVs demonstrating a significant increase in CD31^+^ vessel structures. Notably, the cell spheroid EV group exhibited the most substantial increase in CD31^+^ vessel structures compared to the control group. Although both the cell monolayer and cell sheet EV groups also displayed noteworthy increases, their improvements fell slightly short of the outcomes achieved by the cell spheroid EV group.

For a more detailed quantitative assessment of the CD31^+^ vessel structures within the tissue samples (Fig. 10B), imaging software (ImageJ) was used to perform semiquantitative analysis based on the intensity of the fluorescence signals in each group. The results were illuminating: the control group displayed a CD31 fluorescence intensity level of 4.53 ± 1.1; the cell monolayer group indicated 10.8 ± 2.1; the cell sheet group demonstrated 12.4 ± 2.5, and the cell spheroid group exhibited a remarkable 20.2 ± 3. These values underscored the substantial differences in angiogenesis between the experimental groups, with the cell spheroid EV treatment clearly standing out as the most potent stimulator of new blood vessel formation.

In addition to CD31 immunofluorescence staining, H&E staining was applied (Fig. S3) to delve deeper into the tissue architecture and structure visualization of the various samples. This method served as a corroborating source of evidence, further confirming the trends and outcomes observed in our previous analyses. Comparison of the different histological features of the various experimental groups marked an increase in the presence of angiogenic structures, particularly in the cell spheroid EV-treated samples. This observation lent strong support to the effectiveness of wound healing properties resulting from the influence of the different EV treatments, as the histological features closely aligned with the angiogenic outcomes.

## 4 Discussion

As a part of the broad category of extracellular vesicles (EVs) produced by cells are some of the foremost and critical elements of the paracrine effect produced by MSCs, on account of their substantial reparative achievements, safety, and compatibility with living organisms, in addition to their capacity to be selectively modified to enhance therapeutic effectiveness. Due to these characteristics, they have been long considered favorable candidates for diabetic wound treatments [41,42]. Nevertheless, limitations in obtaining a satisfactory level of yield for these nanoscale vesicles have greatly slowed their usage in clinical settings, as their incorporation into this particular field mandates substantial improvements in the quantities produced. A number of prior studies have reported the application of a variety of techniques and approaches designed to enhance the secretion of EVs, ranging from mechanical to sonic and chemical stress. However, these strategies have yet to achieve a satisfactory translation into clinical usage due to their difficulty in technical application. Therefore, this study puts forward an approach based on the use of three-dimensional spheroid cell culturing techniques on human adipose-derived stem cells (hASC) to enhance the release of EVs, while comparing the effects other similar cell culturing techniques have on the production of EVs with a special focus on the in vivo effects of these in the diabetic rat wound model. The strengths of the various cell culturing techniques rely on the simplicity of their application, as well as their cost-effectiveness and applicability.

Following the fine-tuning of the numerous factors involved in the fabrication of the cell spheroids, it has been found that the use of cell spheroids, both in the literature and our own experiments has been irrevocably related to the increase of many useful characteristics in a great variety of cell lines [43]. In our research, more specifically, spheroids consisting of 4000 cells per spheroid were able to promote hASC to increase EV secretion, stimulating a significant increase in protein quantity, as well as an increase in particle numbers. Meanwhile, the use of the second cell culture technique, cell sheets, resulted in a comparatively diminished increase in both protein increase and concentration, indicating the superiority of the three-dimensional spheroid cell culture. After conducting miRNA-sequence analysis and employing bioinformatics methodologies, it was revealed that extracellular vesicles derived from spheroid-cultured hASC exhibited a substantial enrichment of miRNAs that are closely associated with the wound healing processes. This discovery underscores the potential relevance of these vesicles in wound healing applications, highlighting their enhanced miRNA content compared to other cell culture conditions.

EVs derived from the different cell culture techniques successfully promoted the HS68 fibroblast and HUVEC viability and proliferation in addition to enhancing the formation of tubes in HUVEC. Among the different culture method-derived EVs, the greatest enhancement was observed in the spheroid group. This occurrence was repeatedly observed throughout the various tests, especially in the wound healing test, by promoting cell proliferation, migration, and angiogenesis. Our results indicated that the usage of different cell culture morphologies, such as cell sheets and spheroids, could markedly enhance the quantity of EVs produced by hASC while augmenting their tissue recovery-promoting effects, transforming these EVs into better diabetic wound healing tools.

The variables involved in the formation of the cell sheets and cell spheroids were carefully selected and tested from a combination of previous data obtained and relevant literature. Within the desired framework, the most relevant parameters were the size of the cell spheroids (the number of cells used) and the period of the spheroid culture (the time in which the spheroid remained formed). Regarding the number of cells used, the literature indicates that an increase in cell spheroid size can be correlated to an increase in the production of EVs and that said increase has an upper limit [44]. Considering the findings of various studies, values for the cell spheroid formation initially ranged from 2000 to 8000 cells per spheroid. Through the analysis of EV production, the final value of 4000 cells per spheroid was chosen as cell spheroids formed using a superior number of cells showed no significant improvement in their ability to produce EVs. Moreover, regarding the cell spheroid culture period, a maximum time of 24 h was chosen to avoid disassociation or unintended damage to the cells.

By employing NTA and BCA protein assays, it was found that the use of cell sheets and particularly cell spheroids (formed by 4000 cells for a period of 24 h) markedly enhanced hASC production of EVs quantity. EV production achieved by cell sheets saw an increase of 9 % in particle concentration and 15 % in protein concentration. Meanwhile, cell spheroids experienced an increase of 20 % in particle concentration and 45 % in protein concentration. These increases align with similarly obtained values found by a variety of previous studies and methods such as acoustic stimulation (8-fold) [45], electrical stimulation (1.7-fold) [46,47], molecular inhibition (3-fold) [48,49], small molecular modulation (3-fold) [50], hypoxia exposure (1.3-fold) [[51], [52], [53], [54], [55], [56], [57]], PH level (6-fold) [[58], [59], [60], [61]], hyperglycemia exposure (4-fold) [62], nanoparticle endocytosis (5–20 fold) [63] and mechanical loading (30-fold) [64]. To date, the EV increase obtained from the culture of the cell spheroids previously described has yielded a significantly high increase in the quantities of EVs, comparable to many of the previously mentioned published studies, with considerably simpler setups and procedures.

Characterization of the different EVs (monolayer, sheet, and spheroid) under TEM indicated that all EVs displayed the typical round appearance expected of EVs, along with the appropriate specific marker expression. However, a distinction between the groups was found when characterizing their variations in size with monolayer EVs presenting an average size of 153.5 ± 27 nm, EVs from sheets at 180 ± 30 nm, and EVs from spheroids at 209 ± 38 nm, indicating a correlation between the increase of the EV production and size of these. Furthermore, no significant differences were observed between the cell internalization of monolayer EVs versus spheroid EVs. Consequently, the use of the aforementioned parameters for the fabrication of spheroids was judged suitable for the increase of EV release, especially considering that hASC cells subjected to this cell culture technique and subsequent EV harvesting showed no significant increase in apoptosis, compared to hASC monolayer cultured cells. Although some physical changes were observed post-spheroid culturing, no significant increase in apoptosis was observed in the hASCs following spheroid formation and culturing. Moreover, spheroid EVs exhibited an increase in quantity and improvements in their biological effects. Similar effects have been registered in other studies, particularly those of EVs exposed to hypoxic treatments. Such reports have indicated that extracellular vesicles produced under low oxygen conditions exhibit an enhancement in their innate angiogenic capabilities and diabetic wound healing-promoting effects [[51], [52], [53], [54], [55], [56], [57]].

Along with the physical changes recorded in both the size and yield of EVs derived from the cell sheets and cell spheroids, further changes have been registered at the genetic level in both of these cell groups [32]. Analysis of the expression profiles of genes involved in the biogenesis and the secretion of EVs indicated that a great number of these were significantly enhanced in both of the desired cell morphologies. The various genes involved in the biogenesis and production of EV genes, such as CD82, were increased by a log2-fold change factor of 3.31 (spheroid vs. monolayer), 1.46 (sheet vs. monolayer), and 1.85 (spheroid vs. sheet), which indicated a considerable enhancement on the part of cell spheroids when compared to both cell sheets and cell monolayers. This increase in expression is of great importance as CD82 has displayed a critical role in the final stages of EV formation and release [65,66]. Additionally, RAB7B registered a similar increase by a log2-fold change factor of 2.62 (spheroid vs. monolayer), 1.08 (sheet vs. monolayer), and 1.54 (spheroid vs. sheet). This gene, in particular, has been implicated in the regulation of medium to significant EV release [67]. Meanwhile, an increase in the log2-fold change of the SDCBP gene was also recorded at 1.33 fold (spheroid vs. monolayer). SDCBP has been linked to the regulation and production of EVs through the binding of Syndecan [68]. Moreover, it was noted that there was no significant difference when comparing sheet and monolayer groups, indicating the superiority of spheroid groups in this particular aspect. A final analysis of the genes indicated an increase in the RAB27 A/B log2-fold change of 3.14 (spheroid vs. monolayer), 0.24 (sheet vs. monolayer), and 2.90 (spheroid vs. sheet). This pair of genes has been found to be essential for exosomal release in different cell lines, predominantly found within late endosome processes [69].

Moreover, through Bubble Chart Pathway Enrichment analysis of RNA-seq data, significant enrichment was identified in several key Gene Ontology Biological Processes (GOBP). The “GOBP response to wounding" pathway was highly significant, indicating that many differentially expressed genes (DEGs) in the cell culture spheroid model are involved in the biological response to injury. The “GOBP wound healing" pathway also showed notable enrichment, suggesting DEGs play crucial roles in tissue repair and regeneration. Additionally, the “GOBP platelet activation" pathway was significantly enriched, highlighting the involvement of DEGs in platelet-related activities essential for hemostasis and wound repair. These results collectively emphasize the critical functions of the identified DEGs in mediating responses to wounding, promoting healing, and activating platelets, providing valuable insights into the molecular mechanisms underlying these processes. When analyzed together with our previous results [32] the observed increase in the upregulation of genes associated with EVs in both cellular groups, and particularly pronounced in the spheroid cell group, was consistently aligned with the concurrently measured yield of EVs. Moreover, the heightened yield documented can be intricately linked to the quantified upregulation. This discernible pattern delineates a nuanced relationship, revealing an intricate interplay between the differential upregulation of genes and the parallel amplification of EV production. These findings underscore a complex system within the cultured cells.

To further understand the effects and functionality of the produced EVs, the biological effects of the various EV groups were evaluated through the changes these presented in their miRNA configuration and from these changes monitored for the EV effects (through their cargo miRNAs) on the regulation of cell growth by posttranscriptional inhibition of gene expression. miRNA sequencing was performed to analyze and determine the differences between each of the EV groups and the various miRNA quantified to determine which of these had the greatest quantifiable changes in both comparisons between cell monolayers and cell sheets with cell spheroids. It was then determined that miRNAs showed enrichment in factors related to wound healing in spheroid-derived EVs. Subsequent analysis revealed a significant positive correlation between the most enriched miRNAs and the promotion of wound healing characteristics such as angiogenesis and cell proliferation. MiR-423-5p, the miRNA with the greatest change in our analysis, plays key roles in promoting angiogenesis and cell proliferation. Previous studies have linked its upregulation to hypoxic environments, where decreased oxygen availability enhances miRNA expression [19]. Additionally, miR-16-5p is known to regulate cell proliferation, exert anti-inflammatory effects, and accelerate re-epithelialization in burn wounds [70]. MiR-93-5p has demonstrated effects in enhancing endothelial cell proliferation and tube formation while mitigating inflammation, conditions similar to those in tumor microenvironments and cell spheroids [71,72]. Of particular significance to angiogenesis in wound healing is miRNA-21-3p, which activates PI3K/Akt and ERK1/2 signaling pathways, thereby enhancing angiogenic activity, regenerative responses, and cutaneous repair [73]. Similarly, miRNA-21-5p upregulation promotes angiogenesis through the upregulation of VEGFR and activation of AKT and MAPK signaling pathways [[74], [75], [76]]. Conversely, spheroid-derived EVs displayed reduced levels of certain miRNAs known for their inhibitory effects on wound healing compared to monolayer EVs. For example, miRNA-15a-3p, associated with the NOX5/ROS signaling pathway and damaging ROS release, and miRNA-26a, which increases in response to diabetic wound injury, were both found at lower levels in spheroid-derived EVs [77,78]. Downregulation or inhibition of these miRNA has been related to the decrease of negative effects in wound healing, such as visible wound healing times increase or escalation of negative physiological characteristics. The changes in the various miRNA expression levels between spheroid-derived EVs and monolayer EVs could be the underlying reason for the increase seen in wound healing-promoting effects of the spheroid-derived EVs.

The results of the miRNA sequencing analysis suggest the wound-healing potential of spheroid-derived EVs. Consequently, their biological effects were subsequently examined, confirmed, and measured through in vitro and in vivo experiments.

In vitro analysis of the EVs indicated that spheroid-derived EVs generated a significant enhancement in the cell viability, proliferation, and migration of both fibroblast cells and endothelial cells, with consistent results throughout the various experiments. Furthermore, their capability to accelerate the formation of tubes in endothelial cells was particularly substantial when compared to monolayer cells. Moreover, the nature of this enhancement was discovered to be of a dose-dependent nature, as an increase in the concentration of any of the EV groups resulted in an increase in the enhancement of their properties. Following the confirmation of the biological effects of EVs in vitro, in vivo experiments were carried out to analyze the effects exerted by spheroid-derived EVs in a diabetic rat model. In vivo wound models were subsequently established using rats induced with diabetes to substantiate the wound healing-promoting effects of the various EV groups. The EVs were delivered into the wound area and periphery. Through the monitoring of the wound healing site, it was determined that the healing rate of the wounds was accelerated significantly in all EV groups, with a considerable increase after the use of the spheroid-derived EVs. Consequently, the in vivo experimental results, after a careful analysis of the wound area, revealed that the spheroid-derived EV group achieved the highest degree of wound closure (close to 3 %) after fourteen days and was similarly associated with the shortest wound healing time. Furthermore, histological staining revealed that the treatment with spheroid-derived EVs significantly facilitated processes such as re-epithelialization, collagen synthesis, remodeling, cell proliferation, and angiogenesis, all of which are closely linked to the healing of skin wounds. These findings were consistent with those of our in vitro experiments.

The mechanism by which the EV increase occurred was investigated. A variety of phenomena were suspected to have influenced the increase in EV release, as previous reports on the use of spheroids have indicated [44,71,79], yet the exact mechanism responsible for this increase still remains unknown. The first phenomenon that has been shown to impact the behavior of cells regarding EV production is the appearance of hypoxia and hypoxia-like conditions in the core area of the cell spheroids, as such phenomena have been shown to enhance the secretion of EVs in other cell culture morphologies [29,[80], [81], [82]]. However, although increases in hypoxia have shown effects on the production of EVs, this condition has been shown to have a limited effect on cell spheroids when the size of the cell spheroid increases to a certain level, indicating a yet-to-be-clarified process regarding low oxygen environments. A similar result was observed when analyzing the second phenomenon affecting the secretion of EVs from cell spheroids, mechanical stress, resulting from the aggregation of cells in the cell spheroid matrix. When comparing cell spheroids with a variety of cell densities, low densities presented a slight increase in the production of EVs, while higher levels of cell density in the production of EVs did not increase. This indicated that the stimulation of mechanical stress has an effect on the physiological functions of cells, such as promoting the release of growth factors, cytokines, or other signaling molecules such as EVs. This mechanical stress has a limited effect on the release from the cells [44].

Therefore, we speculated that stimulation resulting from the combination of these two phenomena together with processes occurring at the cellular level are the likely causes of the resulting promotion of hASC EV secretion. Spheroid-derived EVs presented important wound healing-promoting biological activities both in vitro and in vivo, with enriched miRNA levels associated with this wound healing. Nonetheless, the precise functional implications of miRNAs remain elusive, constituting another constraint within this study and a subject for exploration in forthcoming research endeavors.

Regarding the results of this study, two important outcomes were observed. First, to the best of our knowledge, this is the first study to have simultaneously compared the effects different cell culture morphologies (cell monolayer, cell sheet, and cell spheroid) have on hASC for the promotion of EV secretion. Incorporating the optimal stimulation parameters, the secretion efficiency of EVs derived from spheroid cultures exhibited a significant increase of 40 %. Second, in addition to the increased quantity of EVs that were secreted, more importantly, a considerable change was registered in their miRNA content, which was detected and associated with their increased biological activity, and later confirmed through in-depth in vitro and, more importantly, in vivo studies. From these studies, spheroid-derived, EV-enhanced, diabetic wound healing effects were evaluated rigorously and confirmed in a diabetic rat model. These properties were correlated to previous studies delving into the effects of hypoxia on hASC EVs and their enhanced wound-healing properties [[53], [54], [55], [56], [57]]. In summary, this study represents the inaugural report on the promotion of diabetic skin wound healing by EVs derived from hASC spheroids. Crucially, the novel approach for enhancing EV production demonstrated here may have broader applicability in addressing wounds of various tissue types as well as addressing clinical issues associated with ischemia.

## 5 Conclusions

In this study, the EV qualitative and quantitative characteristics of cell spheroids, cell sheets, and cell monolayers were examined using a variety of characterization techniques ranging from NTA to BCA. Moreover, the presence of various EV-related proteins was confirmed via western blot. Following this, the transcriptomic profiles of hASC spheroid, sheet, and monolayer EVs were analyzed by RNA sequencing, which revealed significant differences in the gene expression pattern, with upregulation of angiogenesis-related genes noted primarily on the hASC spheroids. Throughout the analysis of various in vitro cell experiments such as scratch assays, cell proliferation assays, and tube formation assays on various cell lines, the migration, angiogenesis, and proliferation capabilities of the various EV groups were analyzed. These indicated the superior nature of the spheroid EVs when compared to the other EV groups in all angiogenesis-related properties. Additional results from the analysis of in vivo experiments in diabetic rats confirmed the higher potential that spheroid EVs exhibited, from the rate of wound recovery to the percentage of reepithelization and collagen deposition, as well as a significant increase in angiogenesis-related markers such as CD31. The combination of these results indicates the great potential the usage of three-dimensional spheroid cell culture techniques has for the field of EVs and EV-related wound healing.

## CRediT authorship contribution statement

**Edgar Daniel Quiñones:** Writing – original draft, Project administration, Data curation. **Mu-Hui Wang:** Validation, Project administration, Methodology. **Kuan-Ting Liu:** Project administration, Methodology. **Ting-Yu Lu:** Formal analysis. **Guan-Yu Lan:** Formal analysis, Data curation. **Yu-Ting Lin:** Software, Data curation. **Yu-Liang Chen:** Data curation. **Tang-Long Shen:** Conceptualization. **Pei-Hsun Wu:** Formal analysis. **Yu-Sheng Hsiao:** Resources, Methodology. **Er-Yuan Chuang:** Resources, Project administration. **Jiashing Yu:** Writing – review & editing, Investigation. **Nai-Chen Cheng:** Supervision, Resources, Funding acquisition.

## Declaration of competing interest

The authors declare that they have no known competing financial interests or personal relationships that could have appeared to influence the work reported in this paper.

## Data Availability

Data will be made available on request.
