# Supplementary material for: Extracellular vesicles from human adipose-derived stem cell spheroids: Characterization and therapeutic implications in diabetic wound healing
Source: Mater Today Bio. 2024 Nov 8;29:101333. doi: 10.1016/j.mtbio.2024.101333 (PMC11605404; doi:10.1016/j.mtbio.2024.101333)
Supplement: Multimedia component 1 [file mmc1.docx]

Supporting Information

**Extracellular Vesicles from Human Adipose-Derived Stem Cell Spheroids: Characterization and Therapeutic Implications in Diabetic Wound Healing**

*Edgar Daniel Quiñones^1^,* *Mu-Hui Wang^2^, Kuan-Ting Liu^3^, Ting-Yu Lu^4^, Guan-Yu Lan^3^, Yu-Ting Lin^3^, Yu-Liang Chen^3^, Tang-Long Shen^5^, Pei-Hsun Wu^6^, Yu-Sheng Hsiao^7^, Er-Yuan Chuang^8^, Jiashing Yu^1,3*^ and Nai-Chen Cheng^2,9*^*

^1^ Taiwan International Graduate Program, Sustainable Chemical Science & Technology, Academia Sinica, Institute of Chemistry, Taipei 115, Taiwan

^2^ Department of Surgery, National Taiwan University Hospital and College of Medicine, Taipei 100, Taiwan

^3^ Department of Chemical Engineering, National Taiwan University, Taipei 10617, Taiwan

^4^ Program in Materials Science and Engineering, University of California San Diego, CA, 92093

^5^ Department of Plant Pathology & Microbiology, National Taiwan University

^6^ Johns Hopkins Institute for NanoBioTechnology, John Hopkins University Baltimore, MD 21218

^7^ Department of Materials Science and Engineering, National Taiwan University of Science and Technology, Taipei 106, Taiwan

^8^ School of Biomedical Engineering, Taipei Medical University, Taipei 11031, Taiwan

^9^ Research Center for Developmental Biology and Regenerative Medicine, National Taiwan University, Taipei, Taiwan

**Keywords**: Adipose derived stem cell (ASC), cell spheroids, cell sheet, extracellular vesicles, diabetes wound healing.


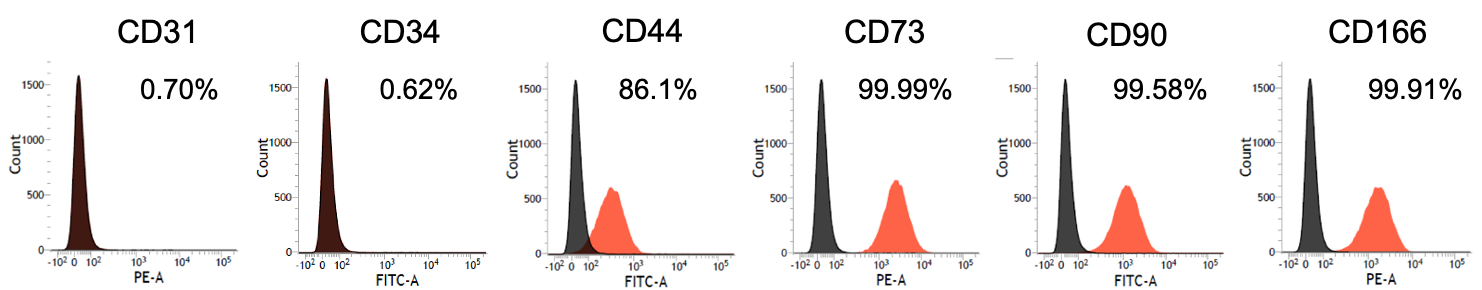


**Figure S1.** Analysis of the surface markers of hASCs with flow cytometry.


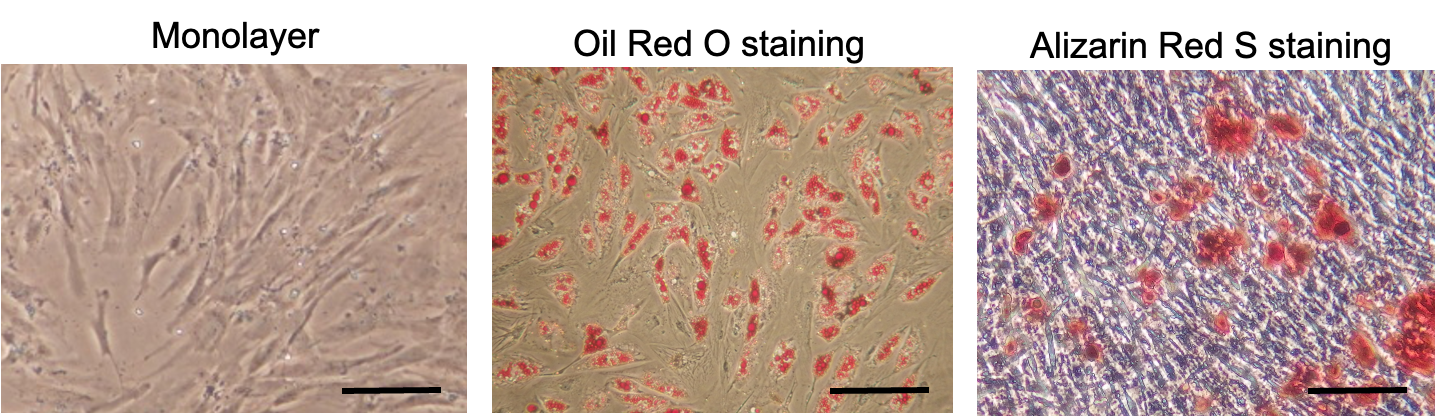


**Figure S2.** hASC oil red O staining and alizarin red S staining.


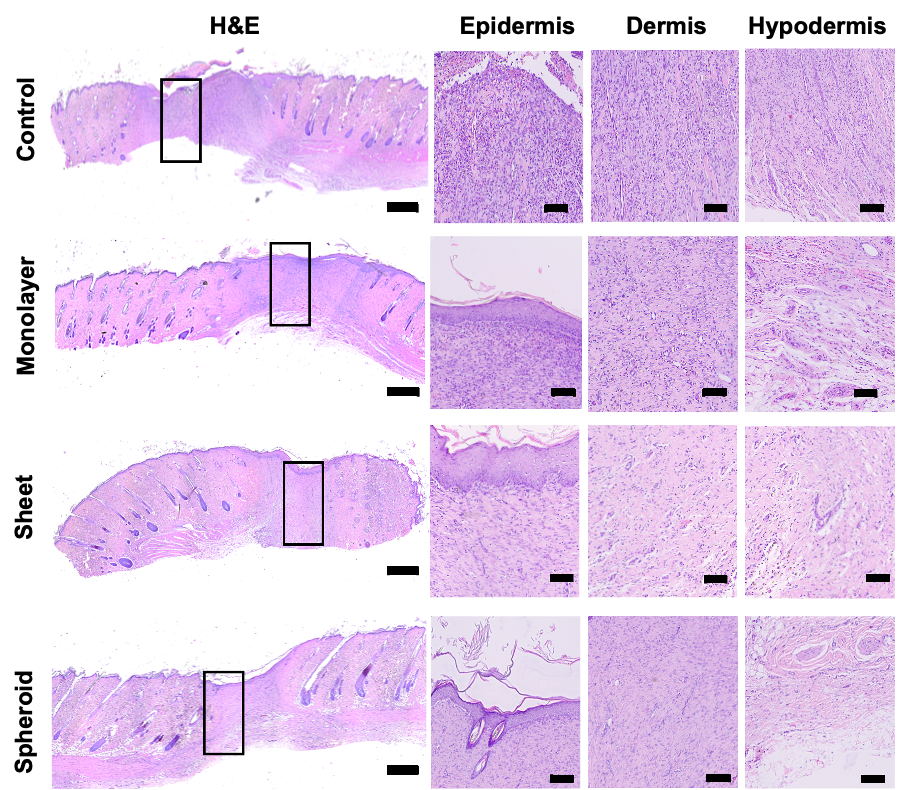


**Figure S3.** H&E staining in the skin wound sections (scale bar: 100 μm and 1000 μm).
